# Dopamine neuron specific RNA-sequencing reveals *Neprilysin 1* acts downstream of the cohesin complex to suppress learning

**DOI:** 10.1038/s42003-026-09690-z

**Published:** 2026-02-16

**Authors:** Illia Pimenov, Courtney M. MacMullen, Chisom Ezeh, Amoolya Sai Dwijesha, Justine David, Akhila Eswaran, Ronald L. Davis, Anna Phan

**Affiliations:** 1 Department of Biological Sciences, University of Alberta, Edmonton, AB Canada; 2 Neuroscience and Mental Health Research Institute, University of Alberta, Edmonton, AB Canada; 3 Women and Children’s Health Research Institute, University of Alberta, Edmonton, AB Canada; 4 Department of Neuroscience, Herbert Wertheim UF Scripps Institute for Biomedical Innovation and Technology, University of Florida, Jupiter, FL USA

**Keywords:** Functional genomics, Classical conditioning

## Abstract

We previously identified Stromalin, a cohesin complex subunit, as a learning suppressor in *Drosophila melanogaster* that acts by limiting synaptic vesicle numbers in dopamine neurons. However, the mechanism by which Stromalin modulates synaptic vesicles remains unclear. We hypothesized that this occurred through the cohesin complex’s function in developmental gene regulation. Through dopamine neuron-specific RNA-sequencing followed by RNAi screening, we identified Neprilysin 1 (Nep1), a zinc-dependent metallopeptidase, to be positively regulated by the cohesin complex and a key downstream effector of Stromalin. *Nep1* knockdown phenocopies *Stromalin* knockdown effects, enhancing learning and memory and increasing synaptic vesicle markers in dopamine neurons. Like *Stromalin*, *Nep1* suppresses synaptic strength between dopamine and mushroom body neurons. Finally, we show *Nep1* overexpression rescues both memory and synaptic vesicle phenotypes caused by *Stromalin* reduction. Interestingly, while cohesin complex appears to set the expression levels for *Nep1* during development, Nep1 function in adult flies supports its learning effects.

## Abstract (web summary)

Dopamine neuron specific RNA-sequencing reveals Neprilysin 1 to be positively regulated by the cohesin complex and a key downstream effector of Stromalin that suppresses learning by limiting synaptic vesicle numbers in dopamine neurons.

## Introduction

Neurobiological and neurocircuit mechanisms supporting learning and memory are exquisitely complicated and tightly controlled. The function of “memory suppressor genes” that act to suppress some aspect of the learning and memory process is one way in which the nervous system exerts this control^1–3^. Thus, removing or reducing the functions of these genes leads to memory enhancements. Memory suppressors have roles during memory encoding, maintenance, and forgetting^2,3^. Although nearly 100 memory suppressor genes have been identified, only a small proportion of these have been studied in depth to reveal how they act to limit learning and/or memory processes^2,3^, significantly adding to our fundamental understanding of memory encoding and storage. For examples, studying the memory suppressors Rac1, a Rho family GTPase, and Scribble, a scaffolding protein connecting Rac1 with downstream signaling molecules, revealed they function downstream of dopamine receptor signaling to promote forgetting^4–7^. Another memory suppressor, choline/acetylcholine transporter SLC22A, was shown to limit acquisition by reducing neurotransmitter signaling^8^. Currently, most memory suppressors identified that act on acquisition appear to function by limiting neuronal excitability or neurotransmitter signaling^2,3^.

We previously identified a novel memory suppressor gene called *Stromalin* in *Drosophila* (homologous to mammalian *Stromalin 1* and *Stromalin 2*), revealing that it acts to limit learning by constraining the number of synaptic vesicles in the dopamine neurons (DANs) required for aversive olfactory memory^9^. This is a highly unusual mechanism, compared with other known memory suppressors^2,3,5,8^. Using serial section electron microscopy and 3D reconstruction, we identified the sizes and morphology of synaptic vesicles (and dense core vesicles) are normal, but there is a 2-fold increase in the numbers of synaptic vesicles when *Stromalin* is knocked down in DANs (controls = 101 synaptic vesicles/μm^3^ neuropil, *Stromalin* KD = 239 synaptic vesicles/μm^3^ neuropil), and a similar doubling was also seen in dense core vesicles (controls = 46 vesicles/μm^3^ neuropil, *Stromalin* KD = 104 vesicles/μm^3^ neuropil)^9^. This resulted in increased dopamine neurotransmitter release and enhanced learning^9^. Moreover, reducing *Stromalin* levels does not change the number and size of synaptic boutons^9^. Little is known about how neurons regulate the numbers of synaptic vesicles that are produced, or the cellular mechanisms leading to their biogenesis in the cell body, in stark contrast to our understanding of synaptic vesicle recycling at synaptic terminals, which is far more advanced^10–12^. Thus, we pursued this effect of *Stromalin* to reveal more about how synaptic vesicle biogenesis occurs and is regulated.

Stromalin, together with SMC1, SMC3, and Rad21, are subunits forming the cohesin complex, canonically known for their role in maintaining sister chromatid cohesion during cell division^13^. However, they also have important roles in regulating gene transcription in cells, including in postmitotic cells^14–20^. The cohesin complex regulates gene transcription through a variety of mechanisms, including through altering three-dimensional DNA architecture, interacting with CCCTC-binding factor (CTCF), Polycomb group protein complexes, or transcription factors^14,15,18,21–23^. Heterozygous mutations in cohesin complex genes, or those of its regulators, result in rare developmental disorders known as cohesinopathies that affect multiple systems, including the nervous system, resulting in cognitive and intellectual disabilities^18,24–27^. The most well-known of these are Cornelia de Lange syndrome and Roberts syndrome^28,29^. Human, mouse, and *Drosophila* cells with such heterozygous mutations generally do not exhibit defects in sister chromatid cohesion^19,30,31^, leading to the speculation that the symptomatic effects of cohesinopathies may result from impairments in gene transcription, rather than defects in chromatid cohesion^14,32,33^. If cohesinopathy symptoms were primarily the result of one or a few dysregulated genes that could be manipulated at later developmental timepoints to attenuate symptoms, then identifying these genes could reveal possible treatment targets even after very early transcriptional programs have been altered.

We hypothesize that Stromalin constrains synaptic vesicle numbers and suppresses learning via transcriptional mechanisms. This is supported by the fact that *Stromalin* knockdown (KD) in postmitotic DANs led to learning enhancements in adult animals and our manipulation did not affect the numbers of DANs^9^. Moreover, these effects of *Stromalin* are likely the result of impairing cohesin complex function, rather than a cohesin-independent effect of *Stromalin*^34^, as *SMC1* KD in DANs also enhanced memory performance, similar to *Stromalin*^9^. Thus, we used cell -specific RNA-sequencing (RNA-seq) to identify the transcriptional differences in 3^rd^ instar DANs when *Stromalin* was knocked down. We then used an RNAi screening strategy to silence differentially expressed genes (DEGs) in DANs to identify which of the transcriptional changes caused learning and synaptic vesicle changes. From these efforts, we identified reduced expression levels of a zinc-dependent metallopeptidase, Neprilysin 1 (Nep1), as the likely regulator of synaptic vesicle numbers in DANs that acts downstream of the cohesin complex. Moreover, reducing *Nep1* expression only in adult flies causes the learning phenotypes. This suggests that even *after* developmental transcriptional programs have been set by the cohesin complex, manipulating *Nep1* levels or function may be able to attenuate some cohesinopathy symptoms.

## Results

### Dopamine neuron specific RNA-sequencing

**Fig. 1:**
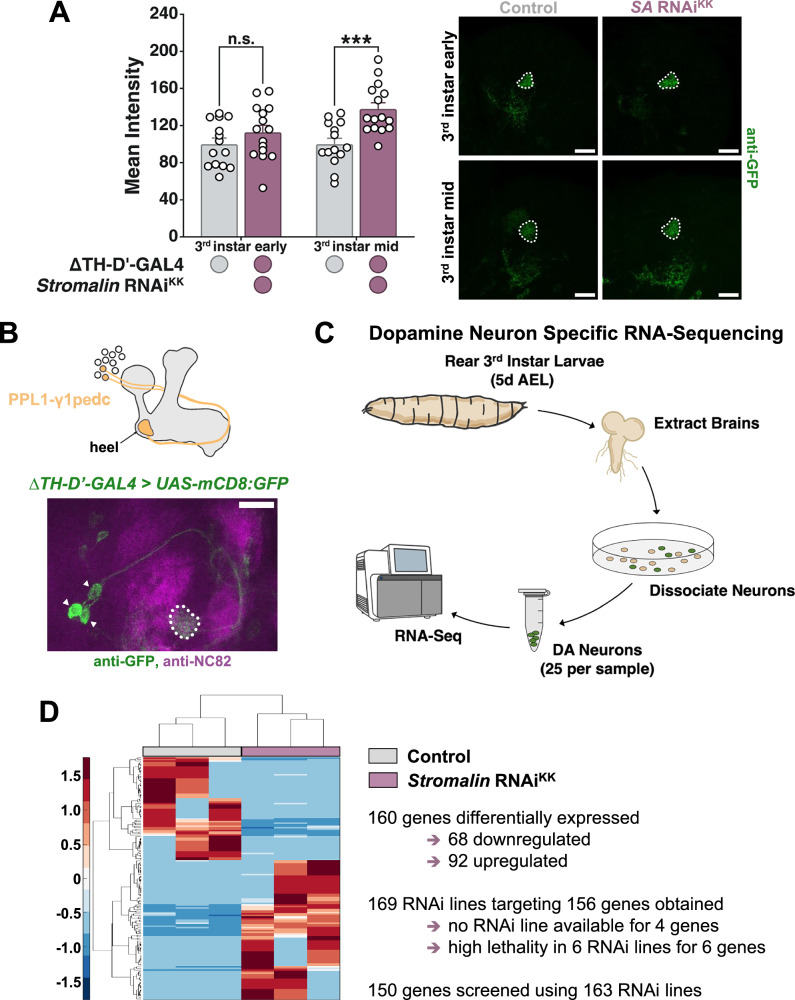
Dopamine neuron specific RNA-sequencing identifies genes dysregulated by *Stromalin* RNAi. **A** *Stromalin* (*SA*) KD in PPL1-γ1pedc dopaminergic neurons (using ∆*TH-D’-GAL4* driver) increases the synaptic vesicle marker Syt:eGFP in the mushroom body heel starting at the middle of the 3^rd^ instar larval stage (3^rd^ instar mid, 5 d AEL). No significant differences were seen at 4 d AEL (3^rd^ instar early). Student’s t test, ***p < 0.001 (*n* = 14,15). All data points were normalized to the control group average (in %). **B** The expression pattern of ∆*TH-D’-GAL4* driver in the 3^rd^ instar larval brain using a membrane marker *UAS-mCD8:GFP*. White arrowheads indicate cell bodies of the PPL1 subset of dopaminergic neurons. **C** GFP-positive PPL1-γ1pedc dopaminergic neurons (25 neurons per sample) were manually collected from 5 d AEL 3^rd^ instar larvae of control and *SA* KD conditions. Larval brains were dissected, their neurons were dissociated, and then the neurons were processed for RNA-sequencing. **D** Clustering and heatmap of significantly differentially expressed genes identified from our RNA-sequencing experiment (log_2_ of normalized gene counts). Relative expression changes are indicated by the color scale (red: high; blue: low). All graphs depict mean + SEM. White dotted lines on micrographs outline the mushroom body heel. Scale bar: 20 µm.

Previously, we demonstrated *Stromalin* functions developmentally to restrict synaptic vesicle (SV) pool sizes, leading to limitations on adult learning^9^. Increases in SV numbers can be observed via increases in the SV marker Synaptotagmin:eGFP (Syt:eGFP) at the synaptic terminals, even when Syt:eGFP is overexpressed (using *UAS-Syt:eGFP*)^9^. Following *Stromalin* RNAi expression in DANs using the tyrosine hydroxylase driver line *TH-GAL4*, Syt:eGFP increases emerge at 5 days after egg laying (AEL; corresponding to the middle of the 3^rd^ instar period), before persisting into adulthood, enhancing cognition^9^. Here, we replicated these Syt:eGFP results using the more restrictive dopaminergic *GAL4* driver, Δ*TH-D’-GAL4* (Fig. 1A). While in adult brains the Δ*TH-D’-GAL4* expresses in all PPL1 DANs that innervate the mushroom body (MB), during the 3^rd^ instar larval stage Δ*TH-D’-GAL4* only expresses in the 2-3 DANs innervating the γ1pedc MB region, known as PPL1-γ1pedc (Fig. 1A)^9^. We chose this driver because we wanted to restrict the heterogeneity of cells for DAN-specific RNA-seq as much as possible, and *Stromalin* KD in the PPL1-γ1pedc is sufficient to enhance learning in adult flies^9^. Additionally, Δ*TH-D’-GAL4* does not strongly express the fluorescent membrane marker mCD8:GFP in any other neurons in the 3^rd^ instar central brain (unlike the *TH-GAL4* driver, which expresses in non-PPL1 dopamine neurons), thus enabling the collection of PPL1-γ1pedc cell bodies for RNA-seq (Fig. 1B). We dissected larval brains from 3^rd^ instar larvae at 5 days AEL, the first time point at which we observe increased Syt:eGFP levels (Fig. 1A). Brains were dissociated and 25 GFP^+^ cell bodies were manually picked per RNA-seq sample (*n* = 3/genotype). RNA was isolated from these cells and subjected to RNA-seq to identify DAN-specific DEGs at the critical time window when SV numbers were increasing in *Stromalin* KD DANs (Fig. 1C). DESeq2 analysis revealed 160 significant DEGs (adjusted p-value; Fig. 1D, Supplementary Data 1). Log_2_ fold change values in general were higher than typical, which is most likely due to the low input RNA levels of our samples. A GO analysis conducted on the 160 significantly dysregulated genes revealed no enriched terms, which may be the result of ‘noise’ due to our low-input RNA samples or our highly restricted sample (dopamine neurons).

### Memory and synaptic vesicle marker screening of differentially expressed genes

To identify which of the DEGs in our DAN-specific RNA-seq dataset might be responsible for *Stromalin’s* effects on memory and SVs, we targeted the DEGs using *Drosophila* RNAi lines and screened them for memory and synaptic protein effects consistent with those of *Stromalin*. We obtained 169 inducible RNAi lines against 156 of the 160 DEGs (2 or 3 RNAi lines were available from one RNAi library for some genes). RNAi lines against 4 genes were not available and thus were not tested: *CR44474, CG6511, Hsc20*, and *l(3)psg2*. The RNAi lines were expressed in DANs using *TH-GAL4* (combined with *UAS*-*dicer2*). While we utilized the Δ*TH-D’-GAL4* driver line to identify PPL1-γ1pedc cell bodies during the 3^rd^ instar larval period, the *TH-GAL4* driver was used for subsequent experiments (unless otherwise stated), since our previous work with *Stromalin* KD revealed stronger phenotypic effects when using *TH-GAL4* than with Δ*TH-D’-GAL4*^9^. Six RNAi lines targeting 6 genes (*Bap55, Pop2, ftz-f1, CG8034, Rpt1*, and *CG3529*) resulted in high embryonic, larval, or pupal lethality, and therefore were not tested in our screens. Thus, 163 RNAi lines targeting 150 genes were tested in a primary memory screen (*n* = 3/RNAi line, control genotypes included at regular intervals throughout).

#### Primary memory screen

**Fig. 2:**
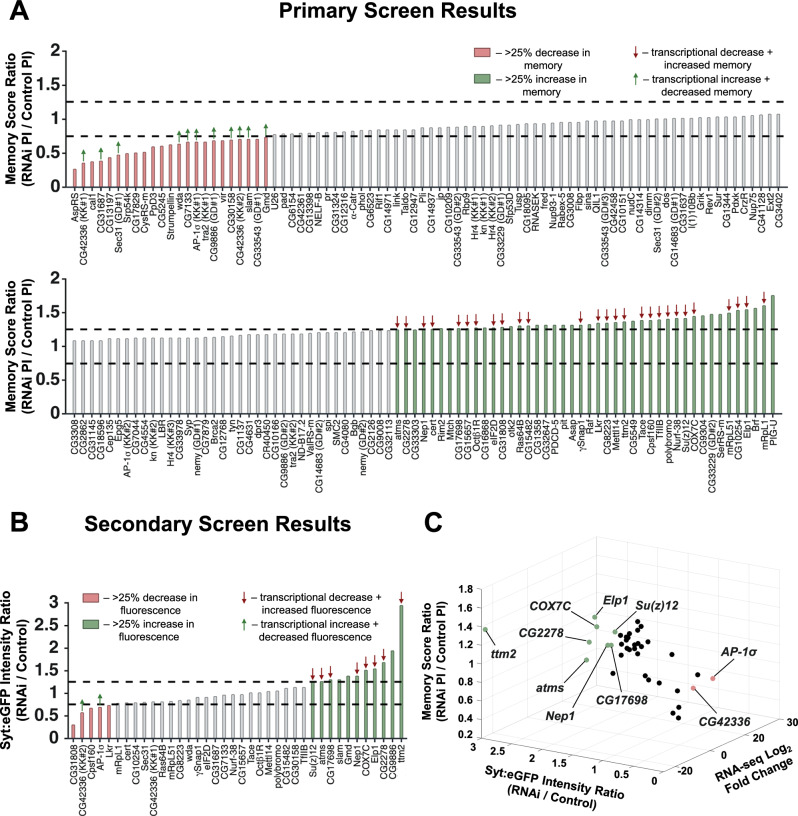
RNAi screens of differentially expressed genes identify gene candidates acting downstream of *Stromalin.* **A** Primary aversive olfactory memory screen results. The bars indicate the intermediate (3 hr) memory score ratios (RNAi PI/Control PI). RNAis were expressed in the dopaminergic neurons using the *TH-GAL4* driver. Red and green bars represent RNAi lines with a > 25% decrease or a > 25% increase in memory scores (relative to control), respectively. Of the RNAi lines that produced effects >±25%, green arrows indicate statistically significantly upregulated genes in *SA* KD cells, and red arrows show statistically significantly downregulated genes in *SA* KD cells from our RNA-sequencing dataset. Black dashed lines denote the thresholds at ±25% of control PI score. **B** Secondary synaptic vesicle marker (*UAS-Syt:eGFP*) screen results. The bars represent Syt:eGFP fluorescence intensity ratios (RNAi/Control). 38 RNAi lines were tested using *TH-GAL4*. Red and green bars represent RNAi lines with a > 25% decrease or a > 25% increase in fluorescence intensity (relative to control), respectively. Of the RNAi lines that produced effects >±25%, green arrows indicate statistically significantly upregulated genes in our *SA* KD cells, and red arrows show statistically significantly downregulated genes in our *SA* KD cells from our RNA-sequencing dataset. Black dashed lines denote the thresholds at ±25% of control Syt:eGFP intensity. **C** A 3D scatter plot of the 37 genes that passed the primary screen plotted along axes representing RNA-seq Log_2_ Fold Change (RNA-seq data), memory score ratio (primary screen results), and Syt:eGFP intensity ratio (secondary screen results). Ten genes that passed the secondary screen are indicated on the plot.

We used liberal criteria for screening to reduce false negatives, reasoning that false positives would easily be detected and eliminated in downstream validation experiments as previously done for a large *Drosophila* RNAi memory screen^35^. The primary memory screen was conducted using an aversive olfactory conditioning assay. Associative memory is tested by pairing one naïve odor with an electric shock while a second naïve odor is not, after which the flies are given a choice test between these two odors in a T-maze. Learning and memory manifest in flies’ avoidance of the shock-associated odor that is quantified as a performance index (PI), with higher PI scores indicating better memory. RNAi lines that produced memory scores (RNAi PI score/average control PI across screen) corresponding to greater than ± 25% change were considered as potentially producing a memory effect, similar to memory screen criteria used previously^35^. From this primary screen, RNAi lines against 10 low and 27 high memory score genes were consistent with the transcriptional and behavioral effects of *Stromalin* KD. Specifically, they displayed either a statistically significant transcriptional increase (positive log_2_FC value from our RNA-seq dataset) along with a low memory score, or a statistically significant transcriptional decrease (negative log_2_FC value from our RNA-seq dataset) along with a high memory score (Fig. 2A and Supplementary Data 2). These 37 genes were tested in a secondary DAN synaptic protein screen, and their mRNA levels were also tested in control and *Stromalin* KD brains using the nCounter system from Nanostring (details below).

#### Secondary synaptic vesicle marker screen

We screened for the effects of knocking down 37 genes on Syt:eGFP levels in DANs (6 brains/RNAi line). We measured fluorescence levels in the γ1pedc region of the MB and considered RNAi lines with fluorescence intensity changes (RNAi fluorescence/control fluorescence) greater than ±25% to potentially produce an SV effect. Of the 10 low memory scoring genes, RNAi lines against 2 genes (*CG42336, AP-1σ*) produced a decrease in Syt:eGFP. Of the 27 high memory scoring genes tested, RNAi lines against 8 genes (*atms, Elp1, CG2278, Su(z)12, CG17698, COX7C, Nep1, ttm2*) produced the expected increase in Syt:eGFP levels consistent with *Stromalin’s* effects (Fig. 2B and Supplementary Data 2). Thus, from our secondary screen, we identified 10 gene candidates that potentially acted downstream of *Stromalin* and the cohesin complex to regulate SV numbers (Fig. 2C).

#### Retesting screen results

**Fig. 3:**
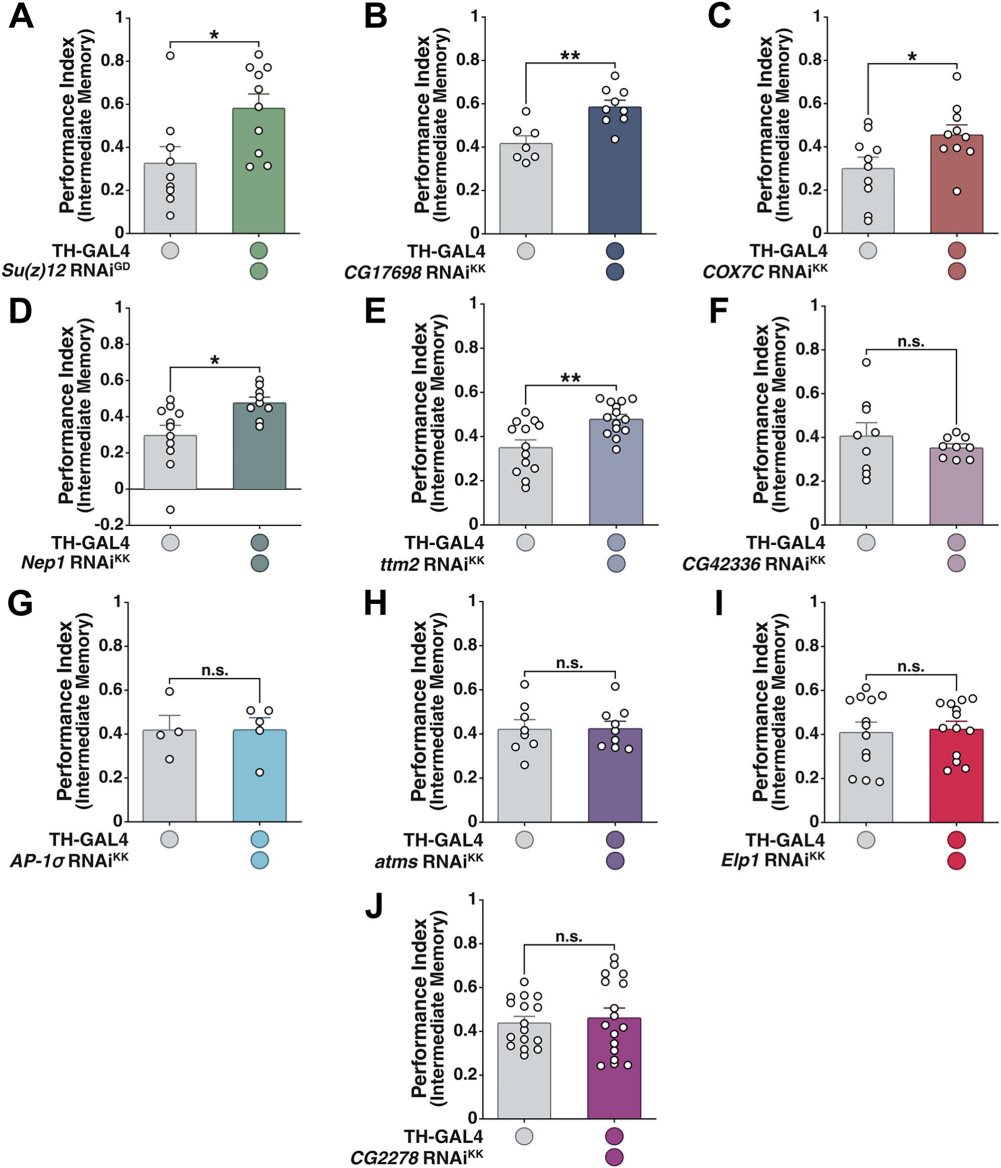
Validation of the primary behavioral RNAi screen results reveals five primary candidates potentially acting downstream of *Stromalin.* **A** *Su(z)12* KD in DANs resulted in significantly higher intermediate memory (3 hr) scores. Student’s t test, *p < 0.05 (*n* = 9,10). **B** *CG17698* KD in DANs enhanced flies’ intermediate memory retention scores. Student’s t test, **p < 0.01 (*n* = 7,9). **C** *COX7C* KD in DANs improved flies’ intermediate memory. Student’s t test, *p < 0.05 (*n* = 10). **D** *Nep1* KD in DANs resulted in significantly increased intermediate memory scores. Student’s t test, *p < 0.05 (*n* = 11,9). **E** *ttm2* KD in DANs resulted in significantly higher intermediate memory scores. Student’s t test, **p < 0.01 (*n* = 13,14). **F** *CG42336* KD in DANs did not change 3 hr memory scores. Student’s t test (*n* = 9). **G** *AP-1σ* KD in DANs did not alter intermediate memory scores. Student’s t test (*n* = 4,5). **H** *atms* KD in DANs did not alter intermediate memory retention. Student’s t test (*n* = 8,9). **I** *Elp1* KD in DANs did not affect intermediate memory. Student’s t test (*n* = 13). **J** *CG2278* KD in DANs did not affect intermediate memory performance. Student’s t test (*n* = 16,17). All graphs depict mean + SEM.

We then attempted to replicate both the behavioral and Syt:eGFP screening results as the first step to eliminate false positives and to validate our gene candidates. Five RNAi lines replicated both their high memory and high SV marker phenotypes seen previously: *Su(z)12, CG17698, COX7C, Nep1, ttm2* (Fig. 3A-J, Supplementary Fig. 1A-1K, and Supplementary Data 2). The other 5 gene candidates failed to replicate either one or both of their memory and Syt:eGFP changes from the screens.

### Verifying transcriptional results from RNA-sequencing

**Fig. 4:**
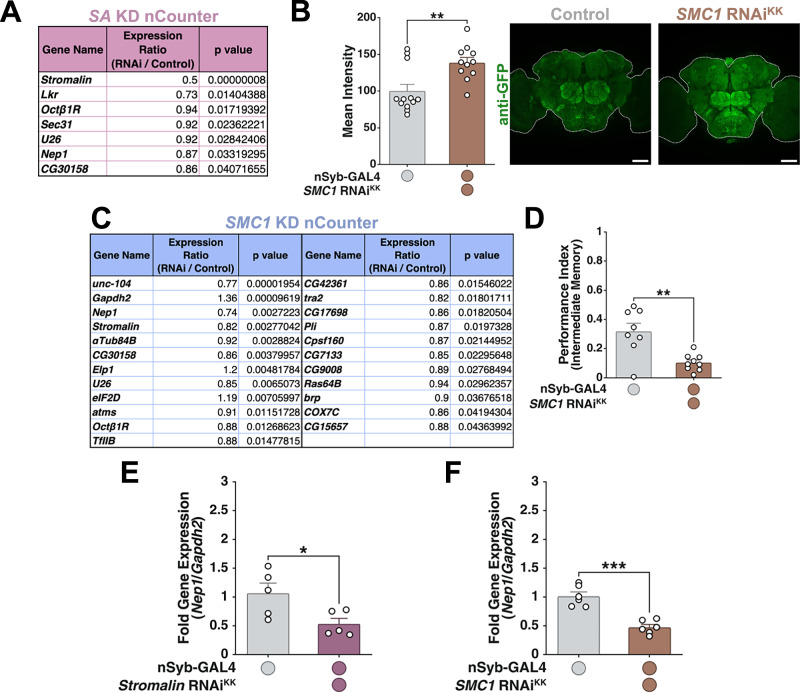
Cohesin complex subunit *SMC1* knockdown phenocopies *Stromalin’s* effects. **A** List of genes having significantly different mRNA levels upon pan-neuronal *SA* KD compared to controls, measured using NanoString nCounter (*n* = 3). Expression was normalized to housekeeping genes *αTub84B*, *Gapdh2,* and to internal housekeeping genes *Act5C*, *brp*, *dcr2*, *unc-104* (highly expressed and unchanged in our RNA-sequencing data). *Lkr*, *Octβ1R*, *Nep1* replicated the downregulation seen in our RNA-seq experiment. Expression ratio indicates the ratio of RNAi group’s average normalized reads to control group’s average normalized reads. NanoString nSolver software was used for data analysis. **B** Pan-neuronal *SMC1* KD increases Syt:eGFP levels in the whole brain of adult female flies. Mann-Whitney U test, **p < 0.01 (*n* = 12,11). All data points were normalized to the control group average (in %). **C** Significantly differentially expressed genes in adult brains with pan-neuronal *SMC1* reduction compared to controls, quantified using NanoString nCounter. Analysis was done in the same way as for *SA* KD nCounter (see Fig. 4A). **D** Pan-neuronal *SMC1* KD impairs intermediate memory retention scores. Student’s t test, **p < 0.01 (*n* = 8,9). **E** RT-qPCR demonstrates *Nep1* expression is reduced when *SA* is knocked down in the whole adult brain, replicating NanoString nCounter results. Student’s t test, *p < 0.05 (*n* = 5). *Gapdh2* was used as a housekeeping gene. Fold gene expression is shown as a $2^{\mbox{-}(\Delta \Delta C_{T})}$ value. **F** RT-qPCR shows *Nep1* expression is also reduced when *SMC1* is knocked down pan-neuronally in adult brains, replicating NanoString nCounter results. Student’s t test, ***p < 0.001 (*n* = 6). *Gapdh2* was used as a housekeeping gene. Fold gene expression is shown as a $2^{\mbox{-}(\Delta \Delta C_{T})}$ value. All graphs depict mean + SEM. White dotted lines on micrographs outline the whole brain. Scale bar: 50 µm.

The results of DAN-specific RNA-seq are difficult to verify due to the very low amounts of RNA recovered from a limited number of DANs. To address this, we used an alternative approach to verify transcriptional differences in our gene candidates. Given that *Stromalin* KD in all neurons enhanced learning and increased Syt:eGFP levels across the whole brain^9^, we reasoned that *Stromalin* likely constrained SVs in a variety of neurons, with effects extending beyond the DANs themselves. Additionally, we reasoned that transcriptional changes occurring during the 3^rd^ instar larval stage likely persist into adult brains, resulting in the adult learning and Syt:eGFP phenotypes. We expressed *Stromalin* RNAi in all neurons (using *nSyb-GAL4*), extracted RNA from adult whole brains, and then used Nanostring’s nCounter system to simultaneously measure mRNA levels of the 37 genes that passed the primary memory screen (Supplementary Data 3). The nCounter system allows for the direct quantification of mRNA without amplification and the resulting reads were quantified, normalized, and differential expression data statistically analyzed using Nanostring’s nSolver program. Our nCounter results confirmed that *Stromalin* mRNA levels were reduced by 50% compared to control brains (Fig. 4A), consistent with the ~50% Stromalin protein reduction previously reported using this same RNAi line^9^. Significant reductions in mRNA levels for 3 genes, *LkR*, *Octβ1R*, and *Nep1* (Fig. 4A), were consistent with our DAN-specific RNA-seq effects. Of these, *Nep1* was the only gene that also passed our RNAi screening experiments.

Knockdown of the cohesin complex subunit SMC1 in DANs also resulted in enhanced memory^9^. Similar to *Stromalin*, we found that *SMC1* KD increases Syt:eGFP levels across the entire adult brain (Fig. 4B). Therefore, we also tested whether whole brain *SMC1* KD affected the expression of the same genes as those affected by whole-brain *Stromalin* KD. Results for 11 genes replicated the DAN-specific RNA-seq data and the direction of the transcriptional effect: *Octβ1R, CG17698, COX7C, Nep1*, *atms*, *TfIIB*, *tra2*, *Cpsf160*, *CG9008*, *Ras64B*, *CG15657* (Fig. 4C and Supplementary Data 4). Of these genes that are transcriptionally dysregulated by *SMC1* KD, *CG17698, COX7C*, and *Nep1* had also passed our screening experiments. Importantly, *Nep1* expression is reduced upon both *Stromalin* and *SMC1* KD. Interestingly, we observed greater transcriptional changes in *SMC1* KD brains compared to *Stromalin* KD brains. This finding is consistent with reports in human patients, where *SMC1* mutations appear to cause more severe symptoms than mutations in *Stromalin 1* or *Stromalin 2*^36–40^. Because *Nep1* showed reduced expression in both our *Stromalin* and *SMC1* KD nCounter experiments, we further verified these *Nep1* expression decreases occurred upon *Stromalin* and *SMC1* KD using RT-qPCR (Fig. 4E, F).

### Eliminating gene candidates as mediators of *Stromalin’s* effects on neurons

**Fig. 5:**
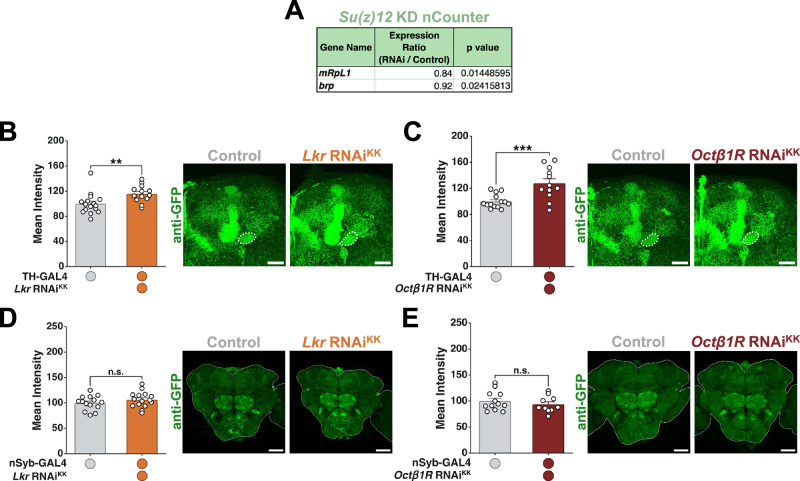
*Su(z)12*, *Lkr*, and *Octβ1R* are unlikely to mediate the effects of *Stromalin* knockdown. **A** Significantly differentially expressed genes in adult brains with pan-neuronal *Su(z)12* reduction when compared to controls, quantified using NanoString nCounter. Analysis was done in the same way as for *SA* KD nCounter (see Fig. 4A). **B** *Lkr* KD in DANs resulted in a significant increase in Syt:eGFP levels in the PPL1-γ1pedc of adult female flies. Mann-Whitney U test, **p < 0.01 (*n* = 16,13). All data points were normalized to the control group average (in %). **C** *Octβ1R* KD in DANs resulted in a significant increase in Syt:eGFP levels in the PPL1-γ1pedc of adult female flies. Student’s t test, ***p < 0.001 (*n* = 14,12). All data points were normalized to the control group average (in %). **D** Pan-neuronal *Lkr* KD did not affect Syt:eGFP levels in the whole brain of adult female flies. Student’s t test (*n* = 14,15). All data points were normalized to the control group average (in %). **E** Pan-neuronal *Octβ1R* KD did not affect Syt:eGFP levels in the whole brain of adult female flies. Student’s t test (*n* = 12,10). All data points were normalized to the control group average (in %). All graphs depict mean + SEM. White dotted lines on micrographs outline the mushroom body heel (B, C) or whole brain (D, E). Scale bar: 20 µm (B, C) or 50 µm (D, E).

The cohesin complex regulates transcription through a variety of mechanisms, one of which is through interacting with Polycomb repressive complexes^23,41,42^. One gene that passed our screening efforts (but failed to replicate its transcriptional downregulation in *Stromalin* KD brains) was *Su(z)12*, a Polycomb repressive complex 2 (PRC2) subunit that regulates transcription. We revisited the possibility that *Su(z)12* might interact with the cohesin complex to regulate SV numbers by determining if *Su(z)12* KD brains could replicate any transcriptional effects seen in *Stromalin* or *SMC1* KD brains. nCounter analysis revealed that whole brain KD of *Su(z)12* significantly dysregulated *mRpL1* and *brp* (Fig. 5A and Supplementary Data 5). Neither of these genes was identified as a candidate from our RNAi screening efforts, nor are they consistent with our *Stromalin* or *SMC1* KD nCounter mRNA data (Fig. 4A, B, Supplementary Data 3, and Supplementary Data 4). Thus, we eliminated *Su(z)12* as a potential mediator of *Stromalin*’s effects on learning and SVs, suggesting the cohesin complex regulates transcription through non-PRC2 mechanisms to regulate synaptic vesicle numbers.

Because *Lkr* and *Octβ1R* mRNA levels were significantly reduced upon *Stromalin* and/or *SMC1* KD (Fig. 4A, C), and they passed the primary memory screen, we revisited the possibility that they were false negatives in the secondary screen. Both *Lkr* and *Octβ1R* KD in DANs increased Syt:eGFP fluorescence in the PPL1-γ1pedc (Fig. 5B, C), phenocopying *Stromalin’s* effects^9^. However, when *Lkr* and *Octβ1R* RNAi lines were expressed pan-neuronally, no changes in Syt:eGFP levels were observed (Fig. 5D, E), whereas pan-neuronal *Stromalin* KD increased Syt:eGFP^9^. Therefore, neither *Lkr* nor *Octβ1R* decreases could fully replicate *Stromalin*’s effects.

The only gene that passed our screening efforts and whose mRNA levels were significantly reduced in both *Stromalin* and *SMC1* KD adult brains was *Neprilysin 1* (*Nep1*).

### *Nep1* phenocopies *Stromalin*’s effects

**Fig. 6:**
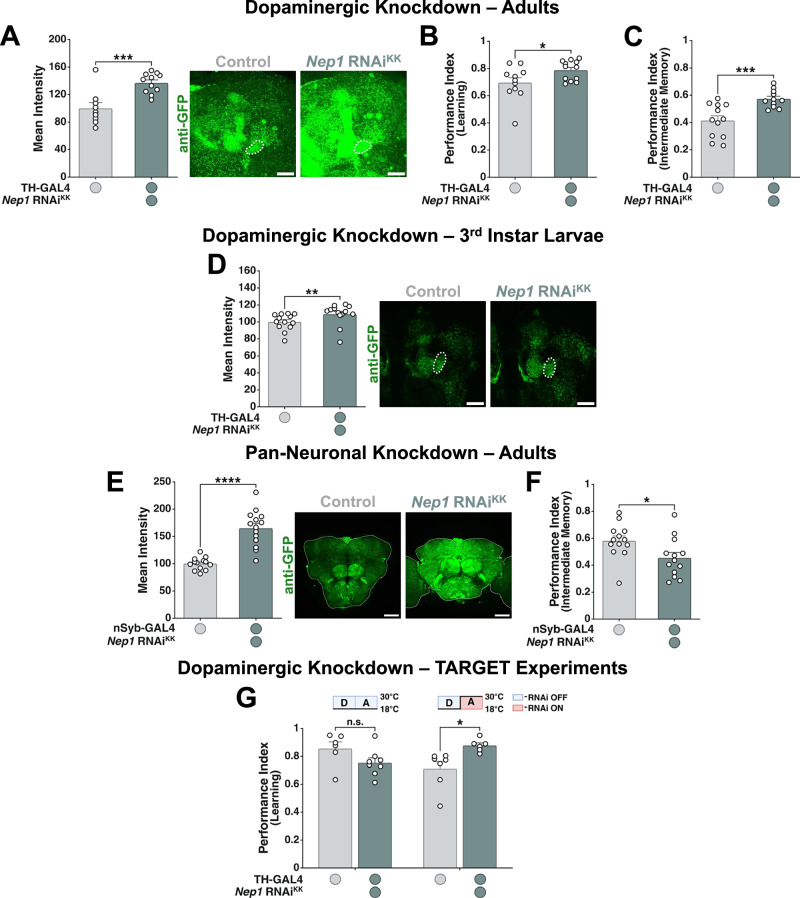
*Neprilysin 1* knockdown phenocopies the effects of *Stromalin* knockdown. **A** *Nep1* KD in DANs of adult female flies increases Syt:eGFP levels in PPL1-γ1pedc. Student’s t test, ***p < 0.001 (*n* = 9,11). All data points were normalized to control group average (in %). **B** KD of *Nep1* resulted in enhanced memory acquisition. Student’s t test, *p < 0.05 (*n* = 11,12). **C** *Nep1* KD in DANs enhances 3 hr aversive olfactory memory. Student’s t test, ***p < 0.001 (*n* = 12,11). **D** Dopaminergic *Nep1* KD increases Syt:eGFP levels in the PPL1-γ1pedc of late 3^rd^ instar larvae. Mann-Whitney U test, **p < 0.01 (*n* = 13). All data points were normalized to the control group average (in %). **E** Pan-neuronal KD of *Nep1* increases Syt:eGFP levels in the whole brain of adult female flies. Student’s t test, ****p < 0.0001 (*n* = 13,14). All data points were normalized to the control group average (in %). **F** Knocking down *Nep1* pan-neuronally decreases 3 hr aversive olfactory memory. Student’s t test, *p < 0.05 (*n* = 13). **G** *Nep1* KD in DANs only during adulthood resulted in enhanced memory acquisition. D – development; A – adulthood. Student’s t test, *p < 0.05 (*n* = 6,8; 7,6). All graphs depict mean + SEM. White dotted lines on micrographs outline the mushroom body heel (A, D) or whole brain (E), Scale bar: 20 µm (A, D) or 50 µm (E).

*Nep1* expression appears to be positively regulated by the cohesin complex in the brain, as KD of both *Stromalin* and *SMC1* reduce *Nep1* mRNA levels in the whole brain (Fig. 4A, C, E, F). Thus, we further investigated *Nep1* to assess how well it phenocopies the effects of *Stromalin* KD observed previously. Reducing *Nep1* in DANs increased Syt:eGFP levels in adult brains (Fig. 6A), and enhanced learning and intermediate memory (Fig. 6B, C), all of which are consistent with the effects of *Stromalin KD* in the brain^9^. Additionally, we observed that the *Nep1* RNAi-induced increase in SV marker levels was also present in the DANs of late 3^rd^ instar larvae (PPL1-γ1pedc; Fig. 6D), similar to what was found with *Stromalin* KD^9^. These findings were also replicated using a second independent RNAi line targeting *Nep1* (Supplementary Fig. 2A-2D). When *Nep1* is reduced in the whole brain, we observed an increase in synaptic protein levels in adult fly brains, similar to the increase observed in *Stromalin* KD brains (Fig. 6E). In contrast to our pan-neuronal *Stromalin* KD findings, pan-neuronal KD of *Nep1* resulted in a decrease in intermediate memory (Fig. 6F), which was consistent with the effect of *SMC1* KD in the whole brain (Fig. 4D). When *Nep1* KD in dopaminergic neurons is restricted to adulthood only using the *GAL80*^*ts*^ TARGET system^43^, we observe an enhanced learning phenotype (Fig. 6G). Thus, *Nep1* in DANs suppresses learning in adults, even though the functions of *Stromalin* (and presumably the cohesin complex) are necessary during development to suppress learning. This is also consistent with the decreased *Nep1* mRNA levels observed in adult brains upon *Stromalin* reduction (Fig. 4A).

### Like *Stromalin*, *Nep1* appears to limit synaptic vesicle numbers

**Fig. 7:**
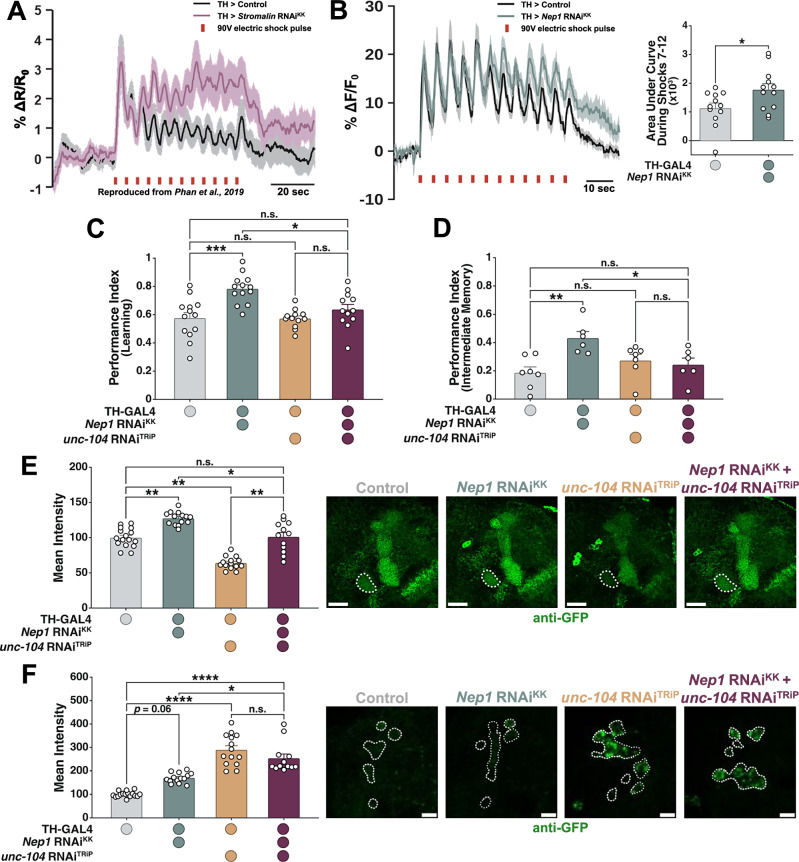
Like *Stromalin*, *Neprilysin 1* appears to limit synaptic vesicle numbers. **A** *SA* KD in DANs resulted in increased cAMP release in response to electric shocks. The mean responses are shown as dark lines and SEM as shaded areas. R = ratio of CFP/YFP fluorescence intensity of cAMP reporter Tepac^vv^ (*n* = 18). Data reproduced and adapted from^9^. **B** Left. Dopamine release in response to 12 electric shock pulses (90 V, 1.25 s each, red bars) measured using GRAB_DA2m_. The mean responses shown as dark lines and SEM as shaded areas. Right. Quantification of second half of shock responses (7-12) as the area under the curve. Mann-Whitney U test, *p < 0.05 (*n* = 12). **C** Concurrent *Nep1* and *unc-104* KD in DANs rescues the learning enhancement of *Nep1* KD only. One-Way ANOVA with Tukey’s *post hoc*, *p < 0.05, ***p < 0.001 (*n* = 13,13,12,12). **D** Concurrent *Nep1* and *unc-104* KD in DANs rescued the intermediate (3 hr) memory enhancement seen in *Nep1* KD flies. One-Way ANOVA with Tukey’s *post hoc*, *p < 0.05, **p < 0.01 (*n* = 7,6,7,6). **E** Simultaneous KD of *Nep1* and *unc-104* in DANs normalized the Syt:eGFP levels in the PPL1-γ1pedc terminals of adult female flies. Kruskal-Wallis test with Dunn’s *post hoc*, *p < 0.05, **p < 0.01 (*n* = 16,14,14,12). All data points were normalized to the control group average (in %). **F** Simultaneous KD of *Nep1* and *unc-104* in DANs, like *unc-104* KD alone, increases Syt:eGFP levels in the cell bodies of PPL1 DANs. Kruskal-Wallis test with Dunn’s *post hoc*, *p < 0.05, ****p < 0.0001 (*n* = 16,14,14,12). All data points were normalized to the control group average (in %). All graphs depict mean + SEM. White dotted lines on micrographs outline the mushroom body heel (E) or cell bodies (F). Scale bar: 20 µm (E) or 10 µm (F).

Previously, we demonstrated *Stromalin* constrained memory by limiting the number of SVs in DANs^9^. *Stromalin* KD produces an increased cAMP signal in the postsynaptic mushroom body neurons (MBNs) in response to dopamine release^9,44^. Since DANs signal the foot-shock aversive stimuli in our memory paradigm (see Methods), this is consistent with the enhanced behavioral memory seen when *Stromalin* is reduced in these neurons. To determine if *Nep1* has a similar function in DANs, we measured shock-evoked dopamine release directly from control DANs and *Nep1* KD DANs using the GRAB_DA_ sensor^45^. GRAB_DA_ is a genetically encoded fluorescent sensor that allows us to measure dopamine release dynamics^45^. When dopamine is released from DANs, it binds to the sensor to increase fluorescence. As previously observed on the postsynaptic side using cAMP imaging (Fig. 7A)^9^, the initial dopamine responses in the MB heel were high in controls, then steadily decreased across the latter half of the 12 foot shocks in the PPL1-γ1pedc compartment (Fig. 7B). However, when *Nep1* was reduced in DANs, the dopamine released after each shock remained consistently high, mirroring the response observed with *Stromalin* KD^9^. This suggests that *Nep1* KD, like *Stromalin* KD, enhances memory acquisition by potentiating shock-induced communication between DANs and MBNs.

The *Drosophila* homolog of the mammalian motor protein *KIF1A*, *unc-104*, is responsible for the anterograde trafficking of SVs from their site of production in the cell body to the synaptic terminal^46,47^. Previously, it was shown that *unc-104* KD was sufficient to rescue learning and SV marker enhancements in *Stromalin* KD flies^9^. Therefore, if *Nep1* constricts SV numbers in the same way as *Stromalin*, we expect *unc-104* KD to also rescue learning and SV marker enhancements in *Nep1* KD flies. While *unc-104* RNAi expression in DANs previously resulted in learning impairments^9^, we did not observe this effect here. Animal behavior can be quite variable and thus, this may have resulted from a number of environmental factors that changed, including our laboratory location. However, the *unc-104* RNAi was still effective at rescuing the *Nep1* RNAi-induced learning and memory enhancements, bringing them back to control levels (Fig. 7C, D). Reducing *unc-104* also normalized the increased Syt:eGFP levels found in PPL1-γ1pedc compartment of *Nep1* KD adult animals (Fig. 7E). Knockdown of *unc-104* alone reduced the Syt:eGFP signal at the synaptic terminal (Fig. 7E), consistent with previous observations^9^. Because *unc-104* traffics SV precursors in an anterograde fashion from the cell body to the axon terminal^46,47^, we also assessed Syt:eGFP expression levels in the PPL1 cell bodies. As expected, control and *Nep1* KD DANs showed low Syt:eGFP fluorescence in the cell bodies, while *unc-104* KD alone, as well as concurrent KD of *unc-104* and *Nep1* resulted in significant increases in SV marker fluorescence in the cell bodies (Fig. 7F).

In summary, our data indicate that both *Stromalin* and *Nep1* act to suppress the synaptic strength between DANs and MBNs.

### *Nep1* is *Stromalin*’s downstream effector regulating learning and synaptic vesicle pool size

Our results suggest that *Nep1* is the most likely candidate to mediate *Stromalin*’s effects on learning and SV pool size. To test this, we assessed whether overexpression of *Nep1* could rescue the effects of *Stromalin* KD on memory and SV marker levels. We used the Nep1^EY21255^ line to overexpress Nep1, as it was previously shown to result in a 2.4-fold increase in *Nep1* mRNA expression when paired with a *GAL4* driver^48^.

**Fig. 8:**
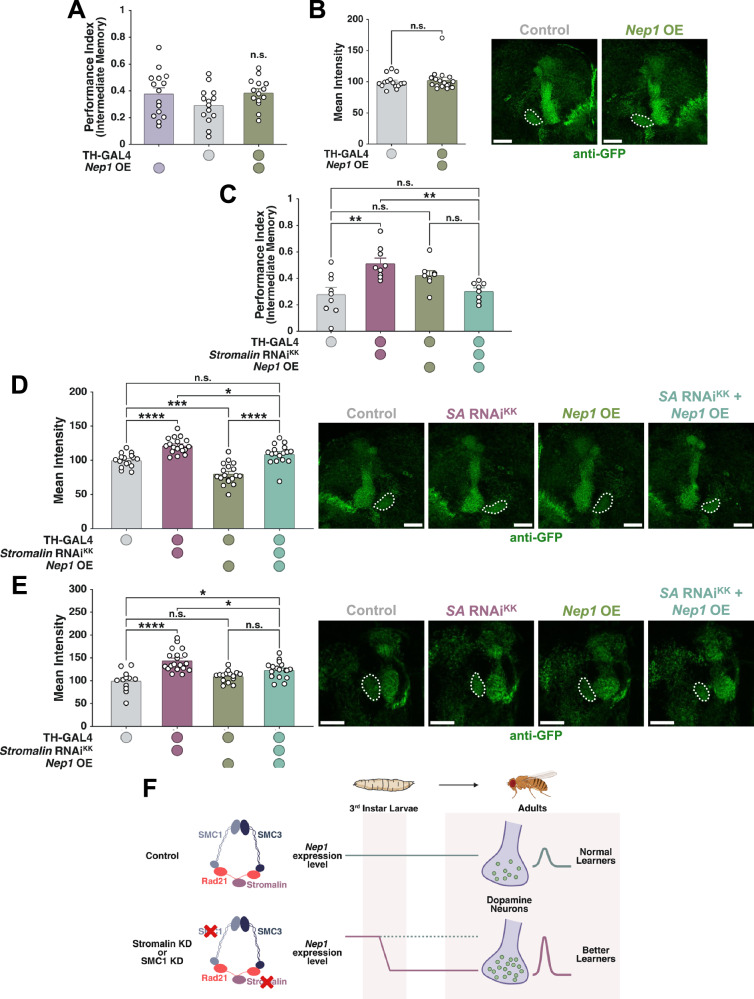
Overexpressing *Neprilysin 1* rescues *Stromalin* RNAi effects on enhanced learning and increased synaptic vesicle numbers. **A** Overexpressing *Nep1* in DANs did not lead to changes in flies’ intermediate memory retention when compared to the genetic controls. One-Way ANOVA with Tukey’s *post hoc* (*n* = 15,14,14). **B** *Nep1* overexpression in dopaminergic neurons did not change Syt:eGFP fluorescence intensity in the PPL1-γ1pedc. Mann-Whitney U test (*n* = 15,17). All data points were normalized to the control group average (in %). **C** *Nep1* overexpression with a simultaneous *SA* KD resulted in a rescue of a memory enhancement caused by *SA* KD alone. One-Way ANOVA with Tukey’s *post hoc*, **p < 0.01 (*n* = 9,9,8,8). **D** *Nep1* overexpression with concurrent *SA* KD resulted in a rescue of a synaptic vesicle pool size enhancement in the PPL1-γ1pedc observed upon *SA* KD alone. One-Way ANOVA with Tukey’s *post hoc*, *p < 0.05, ***p < 0.001, ****p < 0.0001 (*n* = 16,18,18,17). All data points were normalized to the control group average (in %). **E** *Nep1* overexpression combined with *SA* KD in dopaminergic neurons of late 3^rd^ instar larvae resulted in a significant decrease in fluorescence intensity in the PPL1-γ1pedc when compared to *SA* KD alone. One-Way ANOVA with Tukey’s *post hoc*, *p < 0.05, ****p < 0.0001 (*n* = 12,19,15,17). All data points were normalized to the control group average (in %). **F** A summary of the findings presented in this paper. Our current hypothesis is that *Nep1* expression levels are set by the cohesin complex during a previously identified critical developmental window at the 3^rd^ instar larval stage and the expression level changes persist into adulthood, resulting in the learning and synaptic vesicle numbers phenotypes observed. Created in BioRender. Pimenov, I. (2026) https://BioRender.com/6tajfd8. All graphs depict mean + SEM. White dotted lines on micrographs outline the mushroom body heel. Scale bar: 20 µm.

Overexpression of *Nep1* in DANs did not affect intermediate memory (Fig. 8A), nor did it alter Syt:eGFP fluorescence levels in the PPL1-γ1pedc compartment (Fig. 8B). However, when *Nep1* was overexpressed while simultaneously reducing *Stromalin* in DANs, the flies’ memory performance was significantly impaired compared to flies expressing *Stromalin* RNAi alone, and their performance was not different from control flies (Fig. 8C). Similarly, overexpression of *Nep1* while knocking down *Stromalin* also normalized Syt:eGFP levels in the PPL1-γ1pedc compartment of adult brains, making them indistinguishable from controls (Fig. 8D). In this experiment, *Nep1* overexpression alone led to a decreased level of Syt:eGFP, which was not observed in our initial imaging test (Fig. 8B). Since *Stromalin* KD is both necessary and sufficient during the 3^rd^ instar larval stage of *Drosophila* development to elicit its effects on Syt:eGFP and memory in adulthood (Fig. 1A)^9^, we also imaged the Syt:eGFP signal in late 3^rd^ instar PPL1-γ1pedc while overexpressing *Nep1*. Concurrent KD of *Stromalin* with *Nep1* overexpression reduced Syt:eGFP levels compared to the *Stromalin* RNAi-only condition but remained elevated relative to controls (Fig. 8E).

When *Nep1* KD is combined with *Stromalin* KD in DANs, there is an increase in intermediate memory relative to controls that is no different from knocking down either *Nep1 or Stromalin* individually (Supplementary Fig. 3A). This suggests that KD of *Nep1* or *Stromalin* individually is producing a loss-of-function phenotype that increases memory performance to near maximum levels within one particular pathway (increased synaptic vesicles^9^), and therefore simultaneous knockdown of *Nep1* and *Stromalin* does not produce an additive effect. Furthermore, building on our previous findings, we also assessed *Nep1*’s ability to rescue the intermediate memory and SV marker level enhancements in the whole brain. Overexpression of *Nep1* did not affect intermediate memory (Supplementary Fig. 3B) and only trended toward an increase in Syt:eGFP marker levels in adult brains (Supplementary Fig. 3C). However, when *Nep1* overexpression was combined with *Stromalin* KD, we observed a rescue of the memory enhancement in *Stromalin* KD flies, although memory retention was reduced compared with the controls (Supplementary Fig. 3D). By overexpressing *Nep1* alongside concurrent *Stromalin* KD, we were able to reverse the Syt:eGFP marker increase induced by *Stromalin* KD in adult flies (Supplementary Fig. 3E). Thus, *Nep1* overexpression rescues both the memory and SV phenotypes in *Stromalin* KD flies, both in DANs and pan-neuronally.

## Discussion

Through DAN-specific RNA-seq and RNAi screening, we identified *Nep1* dysregulation as being responsible for Stromalin’s effects on learning and SV numbers (Figs. 1–6 and Supplementary Fig. 2). *Nep1* KD phenocopies a variety of *Stromalin* KD effects, including learning and memory enhancements (Fig. 6B, C, and Supplementary Fig. 2A and 2B), increased SV marker levels during development (Fig. 6D and Supplementary Fig. 2D) and adulthood (Fig. 6A and Supplementary Fig. 2C), and potentiates shock-induced communication between DANs and MBNs (Fig. 7B). Finally, *Nep1* overexpression is sufficient to rescue memory and SV marker changes caused by *Stromalin* KD, both in DANs and in whole brains (Fig. 8 and Supplementary Fig. 3B-E). These data support our conclusion that changes in *Nep1* expression levels result in the neurobiological effects of *Stromalin* KD we identified previously (Fig. 8F)^9^. However, it is possible that changes in other genes identified from our screening efforts (*CG17698, Su(z)12, ttm2*, and *COX7C*) may also contribute to the phenotypes observed in one or more neuronal populations.

The reduction of another cohesin complex subunit, SMC1, has similar effects on memory and SV marker levels as *Stromalin* (Fig. 4B)^9^, and *Nep1* mRNA levels are also significantly reduced in *SMC1* KD brains (Fig. 4C, F). Thus, we speculate that *Nep1* dysregulation also occurs when the function of other cohesin complex subunits is impaired or reduced, which should be tested in future studies. Interestingly, expression of RNAi against *SMC1* resulted in greater transcriptional dysregulation in whole brains compared with *Stromalin* RNAi (Fig. 4C). These findings are consistent with reports in patients with cohesinopathies, where individuals with mutations in *SMC1* typically exhibit more severe symptoms than those with mutations in *Stromalin 1* or *Stromalin 2*^36–40^, although for our studies, these differences may arise from the *SMC1* RNAi line possibly having a greater KD efficiency than the *Stromalin* RNAi line. Human patients with mutations in *SMC1, Stromalin 1*, or *Stromalin 2* cohesin complex genes typically exhibit intellectual disabilities of varying severity^36–40^. While whole-brain (pan-neuronal) KD of *Stromalin* results in improved aversive olfactory learning, flies with whole-brain KD of *SMC1* or *Nep1*, and mutant *Nep1* flies, are all impaired in learning and memory (Figs. 4D, 6F, and Supplementary Fig. 2E, respectively), consistent with what is seen in human patients. This could be caused by *Stromalin* RNAi differentially affecting transcription in different subsets of *Drosophila* neurons, leading to stronger *Nep1* transcriptional reductions in DANs to enhance learning, while in other neurons it produces weaker or no effects on *Nep1* transcription. This would also explain why we find less *Stromalin* KD-induced transcriptional changes in the whole brain (Fig. 4A) compared to DAN-specific KD in our RNA-seq (Supplementary Data 1) or whole brain *SMC1* KD (Fig. 4C). Cohesin complex control of gene expression can be highly cell-specific and sensitive to dosage^49^.

While *Nep1* expression levels appear to be set by the cohesin complex during a critical developmental window, this ‘expression level setting’ persists into adulthood to affect adult neural communication. Thus, in theory, increasing *Nep1* expression levels or function in adult *Drosophila* may rescue the effects of reduced cohesin complex function on learning. Interestingly, several RNA-seq experiments conducted in mouse models for cohesinopathies have also identified mouse *Neprilysin* (*NEP*; also known as membrane metalloendopeptidase, *MME*; homologous to *Drosophila Nep1*^50^) as being differentially regulated in their transcriptomic datasets^41,51,52^. Homologs of two other *Drosophila* genes that survived our screening efforts, *CG17689* and *Su(z)12*, also appear in several human or mouse cohesinopathy RNA-seq datasets (homologous to mammalian *CAMKK1/CAMKK2* and *SUZ12*, respectively)^30,41,51,53,54^. Thus, transcriptional changes in *NEP* (or perhaps *CAMKK1/CAMKK2* or *SUZ12*) may also contribute to the phenotypes identified in mouse models, or to cohesinopathy patient symptomatology.

*Drosophila Nep1* is known to be highly expressed in MBNs^55^ (Supplementary Fig. 2F) and was found to support middle- and long-term memory formation in the α/β subset of MBNs^48,56,57^, different from *Nep1* functions in the DANs we observe in this study. How exactly *Drosophila Nep1* functions in DANs or MBNs to affect memory remains unclear. *Nep1* is a membrane-bound zinc metalloendopeptidase known to cleave peptides on the extracellular surface^50^. Neprilysin’s functions were first identified in mammalian brains, where it was shown to cleave several neuropeptides to inhibit their signaling, including enkephalin and tachykinins^58,59^. Thus, one possibility is that reducing *Nep1* expression in *Drosophila* neurons results in increased neuropeptidergic signaling, leading to an indirect increase in synaptic vesicle numbers that may happen as a result of increased neural communication. Alternatively, *Nep1* may cleave other unknown proteins to exert its effects on neural physiology and memory. For example, mammalian *NEP* and *Drosophila Nep1* are known to cleave amyloid-β and their functions have been implicated in attenuating the progression of Alzheimer’s disease^48,60,61^. Thus, it is possible that Nep1 may cleave some proteins that are involved in directly regulating synaptic vesicle numbers or biogenesis. Identification of the genes and molecules that control synaptic vesicle numbers in neurons continues to remain rather elusive. It is possible that some other genes that were identified in our RNA-seq and subsequent screening might have a direct role in regulating the numbers of synaptic vesicles that neurons produce.

## Methods

All data were collected from independent, experimentally naïve animals (no animals were repeatedly tested). Information about fly lines and key reagents used in this study can be found in Supplementary Table 1, Supplementary Data 2, and Supplementary Data 4.

### *Drosophila* Husbandry

*Drosophila* stocks were maintained on standard food at room temperature. Experimental crosses were kept on standard food at 25°C on a 12 hr light-dark cycle. A mix of male and female flies was used for behavioral experiments. For larval imaging experiments, *Drosophila* larvae were not discriminated by sex, whereas only female flies were used for adult imaging experiments. For TARGET experiments (Fig. 6G), flies were reared at 18°C or 30°C, as specified above the graph bars, on a 12 hr light-dark cycle.

### Dopamine Neuron Cell Isolation for RNA-sequencing – Larval Brain Dissection

Several hundred parent flies were crossed and allowed to lay eggs on grape juice agar Petri dishes with active yeast paste for approximately 1.5 hr. These dishes were then discarded. Flies were then allowed to lay eggs for 2 hr on fresh plates. These eggs were then washed with dH_2_O and transferred to vials containing standard fly food medium, and incubated at 25°C. These larvae expressing *UAS-mCD8:GFP*, *UAS-dicer2*, and control or *UAS-Stromalin*^*RNAi*^ transgenes under the control of the ∆*TH-D’-GAL4* driver were then harvested starting at 120 hr AEL at which time their brains were dissected in dissecting saline (9.9 mM HEPES-KOH pH 7.4, 137 mM NaCl, 5.4 mM KCl, 0.17 mM NaH_2_PO_4_, 0.22 mM KH_2_PO_4_, 3.3 mM glucose, and 43.8 mM sucrose; filter sterilized) at room temperature. The ventral nerve cord was manually removed, then brains were placed into cold S2 medium on ice in a low retention 1.5 mL Eppendorf tube. Dissections were limited to under 1 hr by two experimenters, resulting in approximately 140 brains.

### Dopamine Neuron Cell Isolation for RNA-sequencing – Tissue Dissociation

Samples were then centrifuged at 2000 rpm for 1 min, and the S2 medium was removed. The brains were washed three times in dissecting saline with the addition of neural activity inhibitors (20 µM DNQX, 50 µM APV, 0.1 µM TTX). Brains were centrifuged at 2000 rpm for 1 min, then the dissecting saline was removed. A solution of 0.004% (w/v) collagenase (Abnova, cat. #P5279) combined with 5 units of L-cysteine-activated papain in 100 µL volume of dissecting saline (Worthington, cat. #LK003176) was added to the brains and a micropipette set at a volume of 30 µL was used to pipette the brains and solution 30x. The brains were left on a nutator for 20 min at room temperature, pipetting 30x every 5 min. 500 µL of SM active medium (4.18 mM KH_2_PO_4_, 1.05 mM CaCl_2_, 0.7 mM MgSO_4_•7H_2_O, 116 mM NaCl, 8 mM NaHCO_3_, 11.1 mM glucose, 5.29 mM trehalose, 2.4 mM α-ketoglutaric acid, 0.52 mM fumaric acid, 4.47 mM malic acid, 0.51 mM succinic acid, 0.2% (w/v) yeast extract, 20% (v/v) non heat-inactivated FBS, 2 µg/mL insulin, 5 mM Bis-Tris (pH 6.8); solution was adjusted to pH 6.8-6.9 with NaOH and filter sterilized, stored in aliquots at -80°C; inhibitors with final concentrations of 20 µM DNQX, 50 µM APV, and 0.1 µM TTX were added before use) was then added to the brains with papain and collagenase, centrifuged at 2000 rpm for 1 min, and the supernatant removed. The brains were washed 3x in SM active medium. Following the washes, a fresh volume of 500 µL SM active medium was added to the brains. A 1 mL syringe with a 26 G needle was then used to triturate the larval brains until no clumps of tissue were visible, then the solution with dissociated neurons was added to a 30 mm diameter Petri dish containing 750 µL of SM active medium (1.25 mL total) on ice. Cells were left to settle at the bottom of the plate for 30 min.

### Dopamine Neuron Cell Isolation for RNA-sequencing – Neuron Collection

Using a glass pipette with a diameter opening of ~10-20 μm (~2-3x the diameter of our cells) mounted in a micromanipulator and under an inverted fluorescence microscope using a 20x objective, approximately 50-60 GFP^+^ neurons were collected and transferred to a second 35 mm Petri dish containing 1 mL of SM active medium. From this second plate, new glass pipettes were used to collect 25 GFP^+^ neurons while visually excluding GFP^-^ neurons using differential interference contrast (DIC) and fluorescence imaging, ensuring no GFP^-^ cells were included in our sample. The amount of medium carryover was minimized (estimated at <0.5 µL) as the salt in the medium can interfere with the DNase step when preparing libraries for RNA-seq. The 25 GFP^+^ neurons were added to the buffer from a PicoPure RNA isolation kit (Arcturus, cat. #KIT0204). Manufacturer instructions were followed to extract the RNA from the sample, which was eluted in 13 µL of buffer, then stored at -80°C until all samples for the experiment were collected. One 25-neuron sample was collected from one genotype per day, and collections of control and *Stromalin* KD neurons were completed on alternating days, for a total of 3 samples per genotype.

### RNA-sequencing

The NuGEN Ovation SoLo RNA-seq kit with SoLo AnyDeplete Probe Mix for *Drosophila* was used for library preparation. The samples were run on an Illumina NextSeq 500 to obtain approximately 30 million reads per sample using the 2x40bp read chemistry. The results were analyzed by the Center for Computational Biology & Bioinformatics at the University of Florida Scripps Institute for Biomedical Innovation & Technology. The sequence quality for our samples was high as measured by Phred scores were greater than 30. Due to the nature of our samples and low RNA input, RNA quality could not be assessed prior to library creation. However, the number of expressed genes detected in our samples (~2700-4500) were similar or greater than that obtained from *Drosophila* neuron single cell RNA-sequencing experiments (majority of cells ~2000-3400)^62^, which are the most comparable dataset with our dopamine neuron specific RNA-sequencing dataset. Thus, we moved forward with analysis. Transcriptomic data were analyzed using DESeq2.

The RNA-seq data revealed 160 significantly differentially expressed genes between control and *Stromalin* KD samples (adjusted p < 0.05). Log_2_ fold change values are higher than typically found for RNA-seq studies, which are most likely due to the low-input RNA levels of our samples. 92 genes were significantly upregulated and 68 genes were downregulated. We included both up- and downregulated genes in our RNAi screen below, as the cohesin complex is thought to have both enhancer and silencing functions. We looked first for RNAi lines that corresponded to each of these differentially regulated genes in the Vienna *Drosophila* Resource Center (VDRC) RNAi library (preferentially obtaining RNAi lines from the KK library, and if not available, then from the GD library). Five RNAi lines were not available from the VDRC, and thus we obtained them from the Transgenic RNAi Project (TRiP) RNAi library at the Bloomington *Drosophila* Stock Center (BDSC; long non-coding RNA *CR40450, Sfp53D, RNASEK, CG4631, CG31808*). Four genes had no corresponding RNAi line available (long non-coding RNA *CR44474, CG6511, Hsc20, l(3)psg2*) in any of the VDRC or TRiP RNAi libraries. Altogether, we obtained 169 RNAi lines that corresponded to 156 genes identified from our RNA-seq data (due to 2 or 3 RNAi lines being available from one RNAi library for some of our genes of interest).

### RNAi Primary and Secondary Screens

During the RNAi screening process, experimenters were blinded to the RNAi line tested.

The above 169 RNAi lines were crossed with *;UAS-dicer2;TH-GAL4* flies to produce progeny with gene KD in the DANs. 6 RNAi lines resulted in either high embryonic or pupal lethality when crossed with *;UAS-dicer2;TH-GAL4* (*CG3529, CG8034, Bap55, ftz-f1, Pop2, Rpt1*) and therefore were not tested in our screens.

The progeny then underwent a primary learning and memory behavioral screen as adult flies (5-7 days old). We used the olfactory aversive memory assay (3 hr time point^35^), for which methods are described in the “Aversive Olfactory Conditioning” section. We performed the screen with *n* = 4, and then eliminated 1n whose value was the farthest from the mean of each group so that any outliers would not greatly affect the screen results. A control group (corresponding to the appropriate control for the RNAi library used) was included throughout the screen to serve as a reference value. The control groups across the screen were averaged, and this value was used to divide the mean PI scores for each RNAi line (RNAi PI/average control PI across screen). We considered any magnitude greater than ±0.25 to indicate a potential memory effect (corresponding to > 25% change, equivalent to ± ~1 standard deviation, the same criterion used previously for an RNAi memory screen^35^). From this primary screen, RNAi lines against 22 genes (23 RNAi lines) produced low memory scores, while 44 produced high memory scores. Of these, 10 low and 27 high memory score genes were consistent with the transcriptional and behavioral effects of *Stromalin* KD (i.e., statistically significant transcriptional increase and low memory or statistically significant transcriptional decrease and high memory).

These 37 genes (38 RNAi lines) were subjected to a secondary screen by crossing them with *;UAS-Syt:eGFP;UAS-dicer2,TH-GAL4* flies, producing progeny with RNAi and Syt:eGFP expression in the DANs. Six fly (5-6 days old) brains were dissected from each genotype and immunostained for GFP, as we have done for previous experiments^9^, along with a control group (corresponding to the appropriate control for the RNAi library used). The average fluorescence intensity (FI) for the dopaminergic innervation of the mushroom body heel area was calculated. The relative fluorescence intensity (RNAi FI/control FI) was calculated for each RNAi line, and we considered any magnitude greater than ±0.25 to indicate a potential effect on SVs (corresponding to a > 25% change). Of the 10 low memory scoring genes tested, RNAi lines against 2 genes (*CG42336, AP-1σ*) produced a corresponding decrease in Syt:eGFP relative fluorescence. Of the 27 high memory scoring genes tested, RNAi lines against 8 genes (*atms, Elp1, CG2278, Su(z)12, CG17698, COX7C, Nep1, ttm2*) produced a corresponding increase in Syt:eGFP relative fluorescence.

For the 10 RNAi lines that passed both the primary and secondary screen (2 low hits and 8 high hits), we then retested them for 3 hr memory and Syt:eGFP effects. For retesting Syt:eGFP effects, we combined the *UAS-Syt:eGFP* transgenes with the RNAi line, then crossed with *;UAS-dicer2;TH-GAL4* flies to avoid any potential problems resulting from constitutively expressing Syt:eGFP. Five of the 8 high-hit RNAi lines reproduced both significantly higher 3 hr memory scores and higher Syt:eGFP fluorescence intensity compared to controls, while neither of the 2 low hits had scores significantly different from controls. These genes were *Su(z)12, CG17698, COX7C, Nep1*, and *ttm2*.

### NanoString nCounter mRNA Analysis

*Stromalin* RNAi, *Su(z)12* RNAi, or *SMC1* RNAi with their respective control lines were crossed to *;UAS-dicer2;nSyb-GAL4* line to allow for pan-neuronal expression of the transgenes. 5-6 day old adult fly brains (male and female) were dissected in RNAlater^TM^ (Sigma-Aldrich, cat. #R0901) solution and frozen in 1.5 mL test tubes in RNAlater^TM^ at -20°C. 150 brains were dissected per genotype per sample, with 4 biological replicates per experiment. RNA extraction procedure was done 1-2 days after dissections using Qiagen RNeasy^®^ Lipid Tissue Mini Kit (Qiagen, cat. #74804) according to the manufacturer’s protocol and treated with DNase (TURBO DNA-free^TM^ Kit, Invitrogen, cat. #AM1907). RNA quality was assessed (Agilent 2100 Bioanalyzer G2938C), then samples were run using a custom designed probe set on the nCounter system (nCounter Prep Station 5 s & nCounter Digital Analyzer 5 s) at the University of Alberta LMP Pathology Core as per manufacturer’s protocol. RNA was used directly in the nCounter system to quantify mRNA reads (no amplification). As recommended by the manufacturer, we used 6 housekeeping genes for normalization, 2 of which are commonly used for qPCR (*αTub84B* and *Gapdh2*), and 4 genes that were highly expressed and whose expression remained unchanged in our *Stromalin* KD samples, based on our DAN RNA-seq data (*Act5C*, *brp, unc-104*, and *dicer2*; *dicer2* was artificially overexpressed using the *GAL4-UAS* system in our samples). Of note, our *SMC1* and *Su(z)12* samples did show significant gene expression changes in some of the genes designated as housekeeping genes; in future experiments a different set of housekeeping genes should be considered. We used NanoString’s nSolver program to analyze gene expression changes and perform statistical analyses, as per manufacturer’s instructions.

### Reverse Transcription-quantitative Polymerase Chain Reaction (RT-qPCR)

*Stromalin* RNAi or *SMC1* RNAi lines, together with their respective controls, were crossed to *;UAS-dicer2;nSyb-GAL4* line to allow for pan-neuronal expression of the transgenes. 5-7 day old adult fly brains (male and female) were dissected in RNAlater^TM^ (Sigma-Aldrich, cat. #R0901) solution and frozen in 1.5 mL test tubes in RNAlater^TM^ at -20°C. 80-100 brains were dissected per genotype per sample, with 5-6 biological replicates per experiment. RNA extraction procedure was done 1-2 days after dissections using Qiagen RNeasy^®^ Lipid Tissue Mini Kit (Qiagen, cat. #74804) according to the manufacturer’s protocol and treated with DNase (TURBO DNA-free^TM^ Kit, Invitrogen, cat. #AM1907). RNA quality was assessed (Agilent 2100 Bioanalyzer G2938C), then samples were reverse-transcribed with oligo(dT)_20_ primers using the SuperScript^TM^ III First-Strand kit (Invitrogen, cat. #18080-051) according to the manufacturer’s protocol. Amplification was performed using the Applied Biosystems 7500 Fast Real-Time PCR System and 2X SYBR Green qPCR Master Mix (*Dynamite*, a proprietary mix developed and distributed by the Molecular Biology Service Unit (MBSU) in the Department of Biological Sciences at the University of Alberta). Reactions were performed in triplicate. We used *Gapdh2* housekeeping gene for normalization. Fold gene expression is shown as a $2^{\mbox{-}(\Delta \Delta C_{T})}$ value.

### Aversive Olfactory Conditioning

Behavioral experiments were conducted using a standard aversive olfactory conditioning paradigm described previously^35^. Mixed-sex 1-5 day old flies were used for all experiments, unless stated otherwise. The experiments were conducted at 24-26°C under red light, and in ~60-80% humidity. Flies were collected ~24 hr prior to experiments, 60 flies per food vial x 2 for each data point (counterbalancing the trained odor). On the testing day, flies were acclimated for >15 min in a new food vial before aversive olfactory training. Flies were transferred into training tubes where they were exposed to 30 sec of air, 30 sec or 1 min of odor A paired with electric shock (CS^+^), 30 sec of fresh air, 30 sec or 1 min of odor B without electric shock (CS^-^), then 30 sec of air. For learning experiments, flies received 6 shocks across 30 sec while for intermediate memory experiments they received 12 shocks across 1 min, each shock delivered at 90 V for 1.25 sec in duration at 5 sec intervals^9^. Three min or 3 hr after training for learning and intermediate experiments, respectively, flies were placed in a T-maze, where they acclimated for 1 min, then were given 2 min to choose between an arm with the CS^+^ odor and the arm with the CS^-^ odor. Afterwards, the flies were tapped into collection tubes, frozen at -20°C, and counted. A preference index (PI) score was calculated as follows: (CS^-^ - CS^+^)/(CS^-^ + CS^+^). Odors used were diluted in mineral oil (0.05-0.15%), counterbalanced, and pairs of odors were: Benzaldehyde and OCT (3-octanol) that were used for screening, and MCH (4-methylcyclohexanol) and OCT used for all other experiments.

### Immunohistochemistry

Unless otherwise stated, 1-5 day old adult female or larval brains were used for immunohistochemistry experiments. The dissections and processing of whole brains was performed as described previously^63^. Briefly, brains were dissected in Schneider’s Insect Medium (Sigma-Aldrich, cat. #S0146), then fixed with 1% PFA in S2 medium at 4°C overnight. Brains were washed in PAT3 (0.5% Triton X-100, 0.5% BSA in 1x PBS), blocked in PAT3 containing 3% normal goat serum (NGS; 1 hr at room temperature), then incubated with primary antibodies in PAT3 with 3% NGS (3 hr at room temperature, then overnight at 4°C). Brains were washed in PAT3, then incubated with secondary antibodies in 3% NGS in PAT3 (3 hr at room temperature, then for 5 days at 4°C). Brains were washed in PAT3, rinsed quickly in PBS (phosphate buffered saline), then in dH_2_O before mounting between two glass coverslips held apart by spacers on a glass slide. Primary antibodies used include rabbit anti-GFP (1:1000, Invitrogen, cat. #A11122) and mouse monoclonal anti-NC82 (1:25 or 1:50, Developmental Studies Hybridoma Bank, AB_2314866). Secondary antibodies used include goat anti-rabbit Alexa Fluor^TM^ 488 (1:1000, Invitrogen, cat. #A11008) and goat anti-mouse Alexa Fluor^TM^ 633 (1:400, Invitrogen, cat. #A21050).

### Confocal Imaging of Fixed Samples

The brains were imaged on Leica TCS SP5, SP8 or STELLARIS 5 confocal microscope using a 10x, 20x water immersion, or 25x water immersion objective with 488 nm and/or 638 nm laser excitation. All brain images were imaged using 1 µm image stacks. Images were analyzed using ImageJ. For whole brain fluorescence analysis, a region of interest (ROI) was drawn outlining the brain. For measuring the fluorescence in the mushroom body heel, the fluorescence intensity values were obtained by drawing a circular ROI of a consistent size within the heel per experiment. For analysis of dopaminergic PPL1 cell bodies, ROIs were drawn around all visible individual cell bodies. The mean fluorescence intensity values were averaged between the anterior and posterior limits of the structure of interest. Data from both brain hemispheres (for heel and cell body analyses) were collected and then averaged to obtain a single value per region per animal. If the region of interest was obscured (e.g., with debris), a hemisphere (whole brain) was excluded from the analysis. All imaging data, unless stated otherwise, were normalized to the control group and expressed as % of the control value for ease of comparison.

### Functional Imaging

Female flies (5-6 days old) were used for in vivo functional imaging experiments as described previously^7^. Non-anesthetized flies were aspirated and their proboscis was glued with myristic acid. Flies were inserted in a custom-designed platform, then their thorax and head were glued to an opening in the platform using UV glue (Fotoplast^®^ Gel, Dreve #44691). A window in the dorsal cuticle of the fly head was cut using a 23 G syringe needle and physiological saline (124 mM NaCl, 3 mM KCl, 20 mM MOPS, 1.5 mM CaCl_2_, 4 mM MgCl_2_•6H_2_O, 5 mM NaHCO_3_, 1 mM NaH_2_PO_4_•H_2_O, 10 mM trehalose, 7 mM sucrose, 10 mM glucose; pH 7.2) was immediately applied. Forceps were used to remove the cuticle, fat tissue, and trachea on the surface of the brain. Flies were checked for normal activity levels before and after imaging. Flies were then mounted under the 25x water immersion objective on Leica STELLARIS 5. The legs of the fly were positioned on a copper grid used to deliver 12 electric shock pulses (90 V, 1.25 sec duration at 5 sec intervals). Dopamine sensor GRAB_DA2m_^45^ was expressed in the MBNs, and the shock responses in the heel region of one hemisphere of the mushroom body were analyzed. Time series fluorescence images were collected across 2 min 30 sec (45 sec baseline, 1 min shock, 45 sec recovery) using 488 nm laser excitation and a frame rate of 4 Hz using a resonance scanner. Fluorescence intensity changes were calculated as ((F_t_ – F_0_)/F_0_) x 100% (where F_0 _= mean fluorescence across 5 sec prior to foot shock and F_t_ = fluorescence intensity at time t) using ImageJ and MATLAB.

### Statistics and Reproducibility

Data were compiled using Excel and/or MATLAB. Statistical analyses were performed using SPSS Statistics. All tests were two-tailed and α = 0.05. All behavioral experiments were analyzed using parametric statistics, as memory PI scores are normally distributed^35^, whereas imaging experiments were analyzed using parametric or nonparametric tests as appropriate. The exact statistical tests and sample sizes used are listed in the figure legends. The graphs were plotted in Excel, Matlab, or BioRender. Exact statistical analysis values can be found in Supplementary Data 5.

### Reporting summary

Further information on research design is available in the Nature Portfolio Reporting Summary linked to this article.

## Supplementary information

Supplementary Information

Description of Additional Supplementary Files

Supplementary Data 1

Supplementary Data 2

Supplementary Data 3

Supplementary Data 4

Supplementary Data 5

Supplementary Data 6

Reporting Summary

## Data Availability

All data supporting the findings of this paper are available within the paper and its Supplementary Files. Dopamine-specific RNA-sequencing data is uploaded to the GEO database (GSE317817). Data for RNA-sequencing and nCounter experiments are provided in Supplementary Data 1 and Supplementary Data 3. The source data for the graphs in the figures is provided in Supplementary Data 2 and Supplementary Data 6. All data are available from the corresponding author upon reasonable request.
