# Supplementary material for: Dopamine neuron specific RNA-sequencing reveals Neprilysin 1 acts downstream of the cohesin complex to suppress learning
Source: Commun Biol. 2026 Feb 16;9:441. doi: 10.1038/s42003-026-09690-z (PMC13021968; doi:10.1038/s42003-026-09690-z)
Supplement: Supplementary file 1 — Supplementary Information [file 42003_2026_9690_MOESM1_ESM.pdf]

Supplementary Information

Dopamine neuron specific RNA-sequencing reveals *Neprilysin 1* acts downstream of the cohesin complex to suppress learning

Illia Pimenov[1,2,3], Courtney M. MacMullen[4], Chisom Ezeh[1], Amoolya Sai Dwijesha[1,2], Justine David[1], Akhila Eswaran[1,2], Ronald L. Davis[4], Anna Phan[1,2,3*]

[1]Department of Biological Sciences, University of Alberta, Edmonton, AB, Canada

[2]Neuroscience and Mental Health Research Institute, University of Alberta, Edmonton, AB, Canada

[3]Women and Children's Health Research Institute, University of Alberta, Edmonton, AB, Canada

[4]Department of Neuroscience, Herbert Wertheim UF Scripps Institute for Biomedical Innovation and Technology, University of Florida, Jupiter, FL, USA

*Corresponding author: anna.phan@ualberta.ca

List of Supplementary Information

Supplementary Table 1. Key reagents and fly lines used in this study.

Supplementary Figure 1. Validation of the secondary synaptic vesicle protein screen results. Related to Figure 3.
Supplementary Figure 2. A second independent *Nep1* RNAi line and *Nep1* mutant line replicate the initial *Nep1* knockdown effects. Related to Figure 6.
Supplementary Figure 3. *Nep1* acts downstream of *Stromalin* to suppress learning and synaptic vesicle numbers. Related to Figure 8.

Supplementary References

Supplementary Table 1. Key reagents and fly lines used in this study.

All sources and identifiers for *Drosophila* stocks used in our RNAi screen are included in Supplementary Data 2. Genotypes of flies used in each figure are provided in Supplementary Data 4.

REAGENT OR RESOURCE	SOURCE	IDENTIFIER
Antibodies		
Rabbit polyclonal anti-GFP	Invitrogen	cat. #A11122; RRID: AB_221569
Mouse monoclonal anti-NC82	Developmental Studies Hybridoma Bank	RRID: AB_2314866
Alexa Fluor™ 488 goat polyclonal anti-rabbit IgG (H+L)	Invitrogen	cat. #A11008; RRID: AB_143165
Alexa Fluor™ 633 goat polyclonal anti-mouse IgG (H+L)	Invitrogen	cat. #A21050; RRID: AB_2535718
Critical Commercial Assays		
Arcturus™ PicoPure™ RNA Isolation Kit	Applied Biosystems	cat. #KIT0204
Ovation® SoLo RNA-Seq Kit with SoLo AnyDeplete Probe Mix, Drosophila	NuGEN	cat. #0407
RNeasy® Lipid Tissue Mini Kit	Qiagen	cat. #74804
TURBO DNA-free™ Kit	Invitrogen	cat. #AM1907
SuperScript™ III First-Strand Synthesis System for RT-PCR	Invitrogen	cat. #18080-051
Experimental Models: Organisms/Strains		
D. melanogaster: ΔTH-D'-GAL4: ;P{ΔTH-D'-Gal4};	Bloomington Drosophila Stock Center	RRID: BDSC_93704
D. melanogaster: TH-GAL4: w*;;P{ple-GAL4.F}	Bloomington Drosophila Stock Center	RRID: BDSC_8848
D. melanogaster: nSyb-GAL4: y^1w*;;P{nSyb-GAL4.S}	Gift from Julie Simpson	N/A
D. melanogaster: R13F02-LexA: w^{1118};P{GMR13F02-lexA}attP40/CyO;	Bloomington Drosophila Stock Center	RRID: BDSC_52460

D. melanogaster: Control (KK library control): w^{1118};empty VIE-260B landing site;	Vienna Drosophila Resource Center	60100
D. melanogaster: *Stromalin* $RNAi^{KK}$ or UAS-$Stromalin^{RNAi}$: w^{1118};P{KK100690}VIE-260B;	Vienna Drosophila Resource Center	106046
D. melanogaster: *SMC1* $RNAi^{KK}$: w^{1118}; P{KK100055}VIE-260B;	Vienna Drosophila Resource Center	108922
D. melanogaster: *Nep1* $RNAi^{KK}$: w^{1118};P{KK101227}VIE-260B;	Vienna Drosophila Resource Center	108660
D. melanogaster: *CG42336* $RNAi^{KK}$: w^{1118};P{KK109998}VIE-260B;	Vienna Drosophila Resource Center	104546
D. melanogaster: *AP1-σ* $RNAi^{KK}$: w^{1118};P{KK108869}VIE-260B;	Vienna Drosophila Resource Center	107322
D. melanogaster: *atms* $RNAi^{KK}$: w^{1118};P{KK100080}VIE-260B;	Vienna Drosophila Resource Center	108826
D. melanogaster: *CG17698* $RNAi^{KK}$: w^{1118};P{KK114647}VIE-260B;	Vienna Drosophila Resource Center	105884
D. melanogaster: *ttm2* $RNAi^{KK}$: w^{1118};P{KK106063}VIE-260B;	Vienna Drosophila Resource Center	100361
D. melanogaster: *CG2278* $RNAi^{KK}$: w^{1118};P{KK107054}VIE-260B;	Vienna Drosophila Resource Center	100549
D. melanogaster: *Elp1* $RNAi^{KK}$: w^{1118};P{KK100227}VIE-260B;	Vienna Drosophila Resource Center	109402
D. melanogaster: *COX7C* $RNAi^{KK}$: w^{1118};P{KK112622}VIE-260B;	Vienna Drosophila Resource Center	104970
D. melanogaster: *LkR* $RNAi^{KK}$: w^{1118};P{KK102546}VIE-260B;	Vienna Drosophila Resource Center	105155
D. melanogaster: *Octβ1R* $RNAi^{KK}$: w^{1118};P{KK102153}VIE-260B;	Vienna Drosophila Resource Center	110537
D. melanogaster: Control (GD library control): w^{1118};;	Vienna Drosophila Resource Center	60000

D. melanogaster: *Su(z)12* $RNAi^{GD}$: w^{1118};;P{GD15573}v42423	Vienna Drosophila Resource Center	42423
D. melanogaster: Control (TRiP library control, 2nd chr.): y^1,v^1;P{CaryP}$Msp300^{attP40}$	Bloomington Drosophila Stock Center	RRID: BDSC_36304
D. melanogaster: *Nep1* $RNAi^{TRiP}$: y^1,sc*,v^1,sev^{21};P{TRiP.HMC05784}attP40	Bloomington Drosophila Stock Center	RRID: BDSC_64911
D. melanogaster: *Nep1* OE or $Nep1^{EY21255:}$ y^1,w^{67c23},P{EPgy2}$Nep1^{EY21255}$;;	Bloomington Drosophila Stock Center	RRID: BDSC_22465
D. melanogaster: Control (TRiP library control, 3rd chr.): y^1,v^1;;P{CaryP}attP2	Bloomington Drosophila Stock Center	RRID: BDSC_36303
D. melanogaster: *unc-104* $RNAi^{TRiP}$: y^1,sc*,v^1,sev^{21};;P{TRiP.GLC01453}attP2	Bloomington Drosophila Stock Center	RRID: BDSC_43264
D. melanogaster: UAS-mCD8::GFP: y*,w*, P{UAS-mCD8::GFP.L};;	[1]	N/A
D. melanogaster: Syt:eGFP or UAS-Syt:eGFP: w*;P{UAS-syt.eGFP};	Bloomington Drosophila Stock Center	RRID: BDSC_6925
D. melanogaster: Syt:eGFP or UAS-Syt:eGFP: w*;;P{UAS-syt.eGFP}	Bloomington Drosophila Stock Center	RRID: BDSC_6926
D. melanogaster: $GRAB_{DA}$: w*;;PBac{10XlexAop-GRAB(DA2m)}VK00005/TM2	Bloomington Drosophila Stock Center	RRID: BDSC_90880
D. melanogaster: UAS-dicer2: w^{1118};P{UAS-Dicer2};	Vienna Drosophila Resource Center	60008
D. melanogaster: UAS-dicer2: w^{1118};;P{UAS-Dicer2}	Vienna Drosophila Resource Center	60009
D. melanogaster: tub>$GAL80^{ts}$: w*;P{tubP-$GAL80^{ts}$};	[2]	N/A

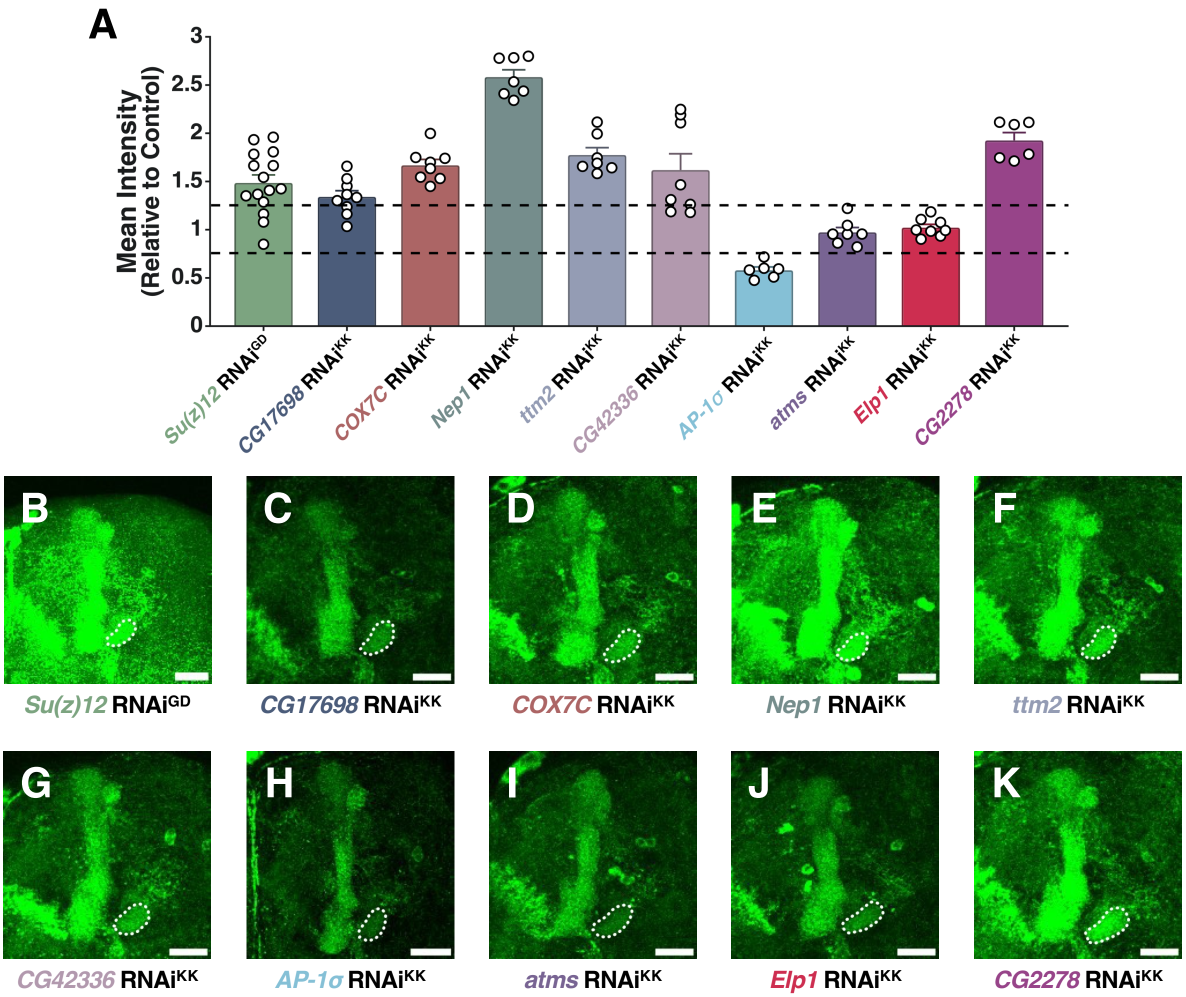

Supplementary Figure 1. Validation of the secondary synaptic vesicle protein screen results. Related to Figure 3.

(A) *Su(z)12* KD, *CG17698* KD, *COX7C* KD, *Nep1* KD, *ttm2* KD, *AP1-σ* KD, and *CG2278* KD replicated Syt:eGFP changes observed during the secondary screen. *CG42336* KD, *atms* KD, and *Elp1* KD did not replicate Syt:eGFP changes initially found during the secondary screen. Black dashed lines denote the thresholds at ±25% of control Syt:eGFP intensity. All data points were normalized to control group average.

Representative images of the adult female mushroom bodies expressing *UAS-Syt:eGFP* and **(B)** *Su(z)12* GD RNAi, **(C)** *CG17698* KK RNAi, **(D)** *COX7C* KK RNAi, **(E)** *Nep1* KK RNAi, **(F)** *ttm2* KK RNAi, **(G)** *CG42336* KK RNAi, **(H)** *AP1-σ* KK RNAi, **(I)** *atms* KK RNAi, **(J)** *Elp1* KK RNAi, **(K)** *CG2278* KK RNAi using *TH-GAL4* driver.

All graphs depict mean + SEM. White dotted lines on micrographs outline the mushroom body heel. Scale bar 20 μm.

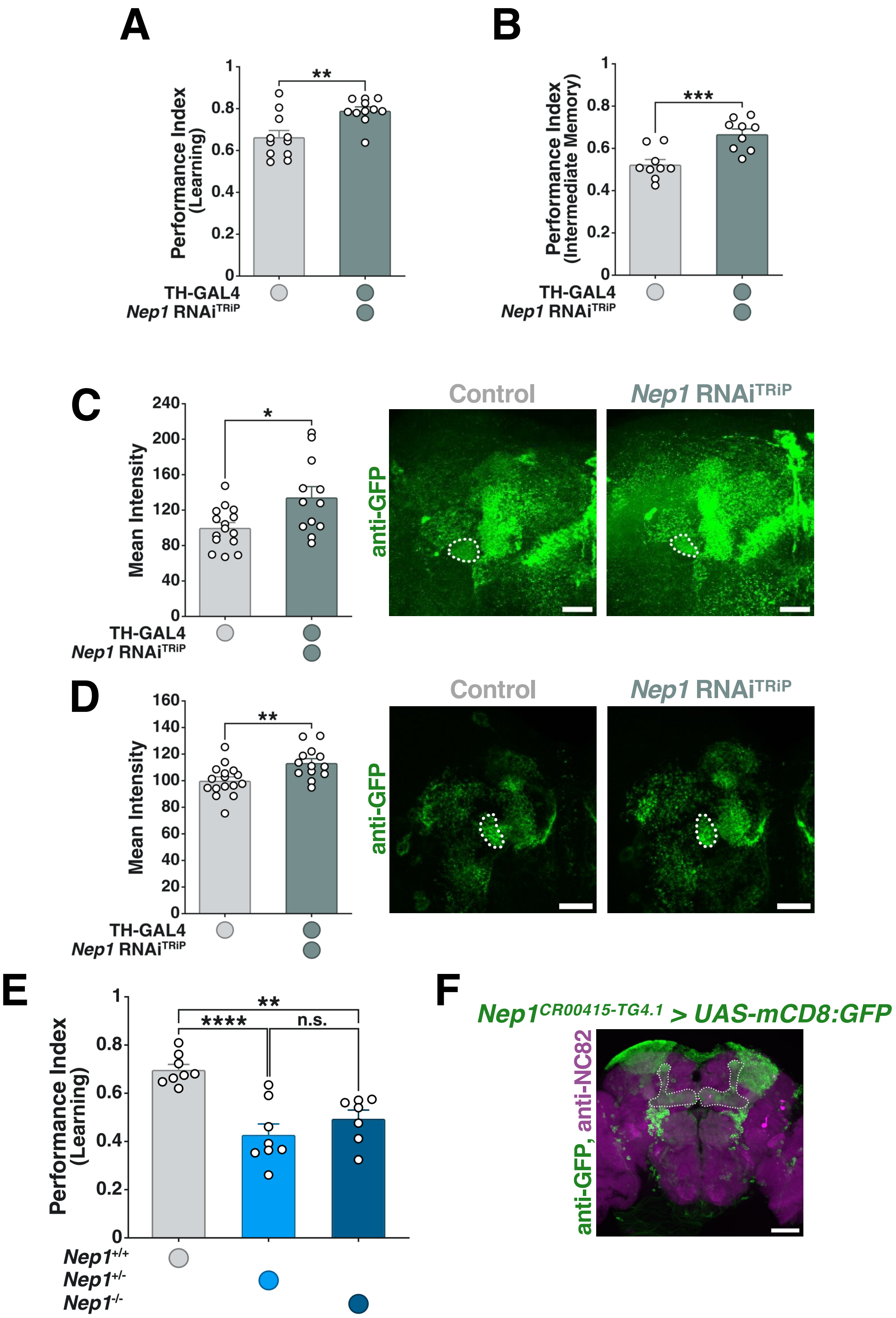

Supplementary Figure 2. A second independent *Nep1* RNAi line and *Nep1* mutant line replicate the initial *Nep1* knockdown effects. Related to Figure 6.

(A) *Nep1* TRiP RNAi KD led to increased memory acquisition scores. Student's t test, $^{**}p < 0.01$ (n=11).

(B) *Nep1* TRiP RNAi KD improved intermediate memory retention scores. Student's t test, $^{***}p < 0.001$ (n=9).

(C) *Nep1* TRiP RNAi KD increased Syt:eGFP levels in the PPL1-γ1pedc of adult female flies. Student's t test, $^{*}p < 0.05$ (n=15,12). All data points were normalized to control group average (in %).

(D) *Nep1* TRiP RNAi KD increased Syt:eGFP levels in the PPL1-γ1pedc of late 3rd instar larvae. Mann-Whitney U test, $^{**}p < 0.01$ (n=16,13). All data points were normalized to control group average (in %).

(E) *Nep1* mutant flies ($Nep1^{CR00415\text{-}TG4.1}$) are impaired in learning, similar to pan-neuronal *SMC1* or *Nep1* KD. One-Way ANOVA with Tukey's *post-hoc*, $^{**}p < 0.01$, $^{****}p < 0.0001$ (n=8,8,7).

(F) *Nep1* is highly expressed in mushroom body neurons as evidenced by fluorescent membrane marker (mCD8:GFP) depicting the endogenous *Nep1* expression pattern (using $Nep1^{CR00415\text{-}TG4.1}$). All graphs depict mean + SEM. White dotted lines on micrographs outline the mushroom body heel (C, D) or whole mushroom body (F). Scale bar 20 µm (C, D) or 50 µm (F).

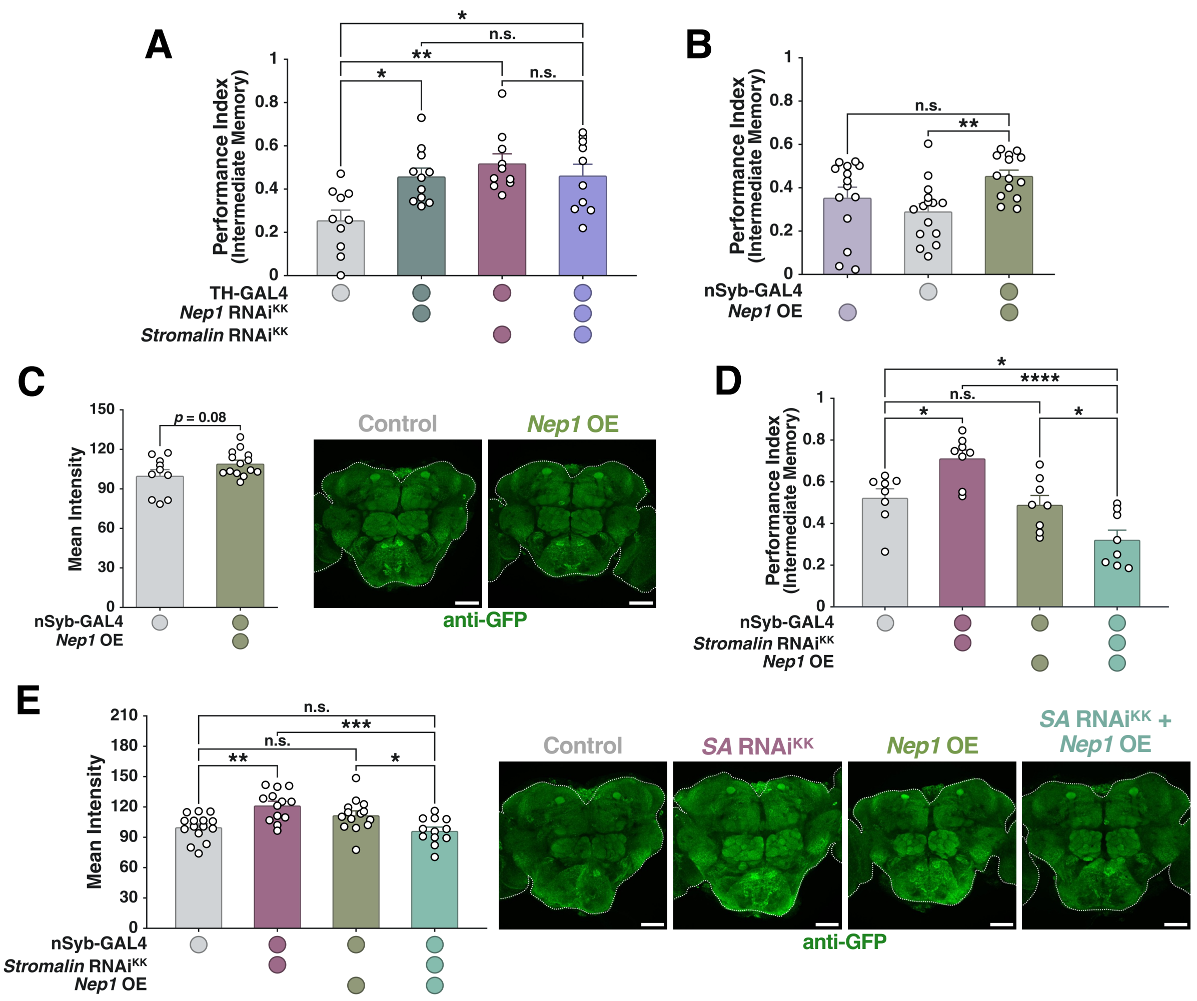

Supplementary Figure 3. *Nep1* acts downstream of *Stromalin* to suppress learning and synaptic vesicle numbers. Related to Figure 8.

(A) *Nep1* KK RNAi KD, *SA* KK RNAi KD, and *Nep1* KK + *SA* KK RNAi KD all result in enhanced intermediate memory retention. Concurrent KD of both *Nep1* and *SA* did not cause more exacerbated increase in memory scores than individual KDs. One-Way ANOVA with Tukey's *post-hoc*, *p < 0.05, **p < 0.01 (n=10,11,10,10).

(B) Overexpressing *Nep1* pan-neuronally did not alter intermediate memory retention scores when compared to the genetic controls. One-Way ANOVA with Tukey's *post-hoc*, **p < 0.01 (n=14,15,14).

(C) Overexpressing *Nep1* pan-neuronally resulted in a trend towards a significant increase of Syt:eGFP fluorescence intensity in the whole brain. Student's t test (n=10,14). All data points were normalized to control group average (in %).

(D) Simultaneous pan-neuronal *Nep1* overexpression and *SA* KD rescues intermediate (3hr) memory enhancement caused by *SA* KD alone. One-Way ANOVA with Tukey's *post-hoc*, *p < 0.05, ****p < 0.0001 (n=8).

(E) Concurrent pan-neuronal *Nep1* overexpression with *SA* KD rescues synaptic vesicle marker increases in the whole brain observed upon *SA* KD alone. One-Way ANOVA with Tukey's *post-hoc*, *p < 0.05, **p < 0.01, ***p < 0.001 (n=15,13,14,12). All data points were normalized to control group average (in %).

All graphs depict mean + SEM. White dotted lines on micrographs outline the whole brain. Scale bar 50 μm.

Supplementary References

1. Lee T & Luo L. *Mosaic Analysis with a Repressible Neurotechnique Cell Marker for Studies of Gene Function in Neuronal Morphogenesis*. (1999).
2. McGuire, S. E., Le, P. T., Osborn, A. J., Matsumoto, K. & Davis, R. L. Spatiotemporal rescue of memory dysfunction in Drosophila. *Science* 302, 1765–8 (2003).
