# Supplementary material for: Dopamine neuron specific RNA-sequencing reveals Neprilysin 1 acts downstream of the cohesin complex to suppress learning
Source: Commun Biol. 2026 Feb 16;9:441. doi: 10.1038/s42003-026-09690-z (PMC13021968; doi:10.1038/s42003-026-09690-z)
Supplement: Supplementary file 2 — Description of Additional Supplementary Files [file 42003_2026_9690_MOESM2_ESM.pdf]

Description of Additional Supplementary File

File name: Supplementary Data 1
Description: RNA-sequencing of 3rd instar PPL1 dopaminergic neurons (control vs Stromalin KD).

File name: Supplementary Data 2
Description: Primary and Secondary Screen data

File name: Supplementary Data 3
Description: nCounter data (Stromalin KD vs control, SMC1 KD vs control, Su(z)12 KD vs control)

File name: Supplementary Data 4
Description: Genotypes of animals in Figures

File name: Supplementary Data 5
Description: Statistical analysis results

File name: Supplementary Data 6
Description: The source data behind the graphs in the paper
